# Characterization of the m^6^A Regulatory Gene Family in *Phaseolus vulgaris* L. and Functional Analysis of *PvMTA* in Response to BCMV Infection

**DOI:** 10.3390/ijms26062748

**Published:** 2025-03-19

**Authors:** Wenyan Wu, Xinhua Wang, Xingrui Liang, Xinqi Huang, Muhammad Amjad Nawaz, Chenchen Jing, Yaru Fan, Jingya Niu, Jing Wu, Xue Feng

**Affiliations:** 1 College of Plant Protection, Shanxi Agricultural University, Jinzhong 030801, China; wwy1112021@126.com (W.W.); wxh13920753409@163.com (X.W.); lxr15383645341@163.com (X.L.); ymynry@163.com (X.H.); jccdyhm@sina.com (C.J.); 16635428702@163.com (Y.F.); jingya1231bts@163.com (J.N.); 2 Advanced Engineering School (Agrobiotek), National Research Tomsk State University, Lenin Ave, 36, 634050 Tomsk, Tomsk Oblast, Russia; m.a.nawaz.cabb.uaf@gmail.com; 3 Centre for Research in the Field of Materials and Technologies, National Research Tomsk State University, Lenin Ave, 36, 634050 Tomsk, Tomsk Oblast, Russia; 4 Institute of Crop Sciences, Chinese Academy of Agricultural Sciences, Beijing 100081, China

**Keywords:** m^6^A, gene family, common bean, BCMV, *PvMTA*

## Abstract

Common bean (*Phaseolus vulgaris* L.) is known for its high protein, dietary fiber, and various trace element contents, making it a widely grown leguminous crop globally. The bean common mosaic virus (BCMV) poses a significant threat to leguminous crop production, causing substantial yield reductions when common beans are infected. Widely occurring in mRNA, the m^6^A modification is vital for maintaining mRNA stability, facilitating splicing, enabling nuclear export, supporting polyadenylation, and initiating translation. Recent studies have identified the m^6^A regulatory gene family in various plant species, and its ability to regulate plant virus infection has been confirmed. There is currently insufficient information regarding the m^6^A regulatory gene family in beans and how it responds to BCMV infection. Consequently, we carried out a genome-wide characterization of the m^6^A regulatory gene family in common bean, which led to the identification of 31 potential regulatory gene members associated with m^6^A. According to evolutionary analysis, the increase in the bean m^6^A regulatory gene family appears to be linked to either whole-genome duplication or segmental duplication events. Subsequent investigations into the expression levels of these genes throughout different phases of BCMV infection showed that all candidate genes responded to the infection with various changes in expression. Moreover, we characterized the methyltransferase activity of PvMTA and validated the interactive relationship between mRNA adenosine methyltransferase A (MTA) and mRNA adenosine methyltransferase B (MTB) in common beans. Through overexpressing and silencing *PvMTA*, we further ascertained that this particular gene has a detrimental impact on the regulation of BCMV infection. This research provides fresh perspectives on the molecular processes that govern the interaction between the common bean and BCMV and aids progress in molecular bean breeding.

## 1. Introduction

Common bean (*Phaseolus vulgaris* L.) is the most widely cultivated edible bean, covering the largest cultivation area and being consumed by the greatest number of people globally. Common bean is characterized by its high protein content, moderate starch level, low fat, and rich nutritional profile, making them an essential source of plant-based protein for humans. They are easily absorbed by the body and can be consumed in large quantities as a staple food [1]. Bean common mosaic virus (BCMV) is one of the most destructive pathogens affecting bean crops. Infection with BCMV can result in various symptoms, including stunting, mosaic, leaf deformation, and even necrosis, which can lead to complete crop failure in severe cases [2,3]. Furthermore, BCMV has a very high seed transmission rate, significantly contributing to its widespread dissemination worldwide [2]. Currently, the control of this virus primarily depends on resistance breeding. The resistance of beans to BCMV is governed by the *I* gene and several recessive resistance genes, including *bc-u* (now referred to as *bc-u*^d^), *bc-1*, *bc-2*, *bc-3*, and *bc-4*. Based on the biological responses of different BCMV strains on bean differential hosts with various genetic backgrounds, these strains are classified into eight pathotypes (PGs), ranging from PG-I to PG-VIII [4]. Because of the complex genetic diversity of BCMV, reports have emerged of strains that can overcome existing resistance [5]. Therefore, it is imperative to identify and develop new sources of resistance. The m^6^A modification plays a vital role in controlling several key agronomic traits in plants. It is also involved in responding to biotic and abiotic stresses, such as infections from viruses [6,7] and bacteria [8], along with unfavorable environmental conditions like drought [9], high salt levels [10], and cold weather [11]. It serves as an excellent starting point for mining potential targets for resistance resources.

N^6^-methyladenosine (m^6^A), which involves methylation at the sixth nitrogen of adenosine, represents the primary internal chemical modification in eukaryotic messenger RNAs (mRNAs) [12]. Recent studies have shown that m^6^A modification also occurs in non-coding RNAs, like circular RNA (circRNA) [13] and long non-coding RNA (lncRNA) [14]. In mammals, this reversible post-transcriptional modification is introduced by a complex of enzymes known as a writer complex, including METTL3 and METTL14, which together make up the catalytic core of the m^6^A methyltransferase [15,16], and WTAP, KIAA1429, HAKAI, RBM15 and its paralog RBM15B, along with the newly identified ZC3H13 [17,18,19,20,21]. This modification can be erased by FTO (fat mass and obesity-associated protein) [22] and ALKBH5 (α-ketoglutarate-dependent dioxygenase) [23]. Reader proteins, particularly those featuring YTH domains, can specifically recognize transcripts that carry the m^6^A modification. A growing body of evidence underscores the essential biological roles of m^6^A. This modification exerts its biological functions by influencing downstream RNA metabolism, encompassing aspects such as mRNA stability, splicing, polyadenylation, translational efficiency, and nuclear export, through the recruitment of reader proteins [24,25,26].

The m^6^A methyltransferase complex in plants has been systematically characterized in *Arabidopsis*, paralleling findings in mammalian species. This complex includes two core methyltransferases: MTA (ortholog of METTL3) and MTB (ortholog of METTL14), along with accessory proteins like FIP37 (WTAP ortholog), VIR (VIRMA/KIAA1229 ortholog), and HAKAI [27,28,29,30,31]. The absence of any of these components leads to reduced m^6^A modification levels across the transcriptome. ALKBH5-related proteins, which belong to the ALKBH family (specifically ALKBH1B through ALKBH13B), primarily facilitate the removal of m^6^A from plant RNAs. This family boasts several to dozens of members in diverse plants, a number that markedly supersedes that observed in animals [32,33]. The most prominent m^6^A readers in plants are proteins that possess the YTH domain, which retains conserved C-terminal region (ECT proteins) [34] and CPSF30 [35].

In the context of plant–virus interactions, several studies have demonstrated that m^6^A modification plays a crucial regulatory role. Martínez-Pérez et al. discovered that the genome of the alfalfa mosaic virus (AMV) contains m^6^A modifications. In *Arabidopsis thaliana* infected with AMV, the knockout of the m^6^A demethylase *AtALKBH9B* resulted in a reduction in viral accumulation compared to wild-type plants, while the m^6^A levels in the AMV genomic RNA increased. This suggests that m^6^A modification may negatively regulate viral infection [6]. Silencing the m^6^A methyltransferase genes *LsMETTL3* and *LsMETTL14* in the planthopper led to a significant increase in the titer of rice black-streak dwarf virus (RBSDV) in the insect’s midgut cells, indicating that m^6^A modification influences RBSDV replication within the planthopper [36]. MeRIP sequencing and subsequent validation experiments revealed that the pepino mosaic virus (PepMV) RNA also exhibits a significant m^6^A modification. Furthermore, the overexpression of m^6^A methyltransferases MTA and HAKAI significantly increased the level of m^6^A modification’s viral RNA, thereby inhibiting PepMV infection [37,38]. Additionally, the PepMV-encoded RNA-dependent RNA polymerase (RdRp) protein can interact with HAKAI and activate the autophagy pathway to degrade the HAKAI protein, thereby disrupting the function of the plant m^6^A methyltransferase complex and protecting the viral genome from methylation damage [37]. After infection with the wheat yellow mosaic virus (WYMV) in both resistant and susceptible wheat varieties, significant differences were observed in the m^6^A modification sites and their abundance on genes related to plant defense responses and plant–pathogen interactions. This suggests that variations in m^6^A modification may contribute to the traits of plant resistance and susceptibility [7]. Further studies revealed that the WYMV-encoded replicase NIb could induce the nuclear export of the wheat methyltransferase TaMTB, which subsequently methylates specific sites on viral RNA1, thereby enhancing the stability of the viral RNA [7]. The m^6^A-seq analysis of rice plants infected with the rice stripe virus (RSV) and the rice black-streaked dwarf virus (RBSDV) demonstrated that the abundance of transcriptome m^6^A modifications increased during viral infection. This included several genes associated with antiviral pathways, such as RNA silencing, disease resistance, and pathways related to antiviral hormones [39]. A comparison of the m^6^A differential peaks following infection with the two viruses revealed that the distribution of m^6^A peaks on the same gene varied, which may explain the different antiviral mechanisms observed in rice plants infected with RSV and RBSDV.

This study aimed to identify m^6^A regulatory genes in common beans and investigated their involvement in viral infections. The research conducted a systemic analysis of the m^6^A regulatory gene family in *P. vulgaris*, encompassing evolutionary and structural analysis, and examined gene expression patterns post-viral infection. The findings showed that multiple members of the m^6^A regulatory gene family contributed to the response to BCMV infection. Notably, MTA, a crucial element of the m^6^A methyltransferase complex in common beans, showed a counteractive regulatory impact on bean common mosaic virus infection. This study advances our comprehension of the critical regulatory role of m^6^A in interactions between beans and BCMV. Investigating m^6^A modifications offers a fresh perspective on plant epigenetic regulation, presenting valuable opportunities to enhance agronomic traits and advance molecular breeding for improved plant resistance.

## 2. Results

### 2.1. Genome-Wide Identification and Characterization of m^6^A Regulatory Genes in Phaseolus vulgaris L.

We identified 36 potential m^6^A regulatory gene candidates in *Phaseolus vulgaris* L. Of these, three lacked the 2OG_Fe(II)_Oxy domain (PF13532), and two more did not contained the YTH domain (PF04146), leading to their exclusion. The transcript ID of each identified gene can be found in Table S1. Based on these results, the bean’s genes were named according to their similarity with their cognate genes in *Arabidopsis* (Table 1). Of note, the common bean genome contains single copies of PvHAKAI, PvFIP37, and PvVIR, which are thought to function as enzymatic subunits present in the m^6^A methyltransferase complex. In contrast, the families of proteins known as ALKBH, MT-A70, and those with YTH domains, were found to be multi-membered.

### 2.2. Chromosomal Location and Collinearity Analysis of m^6^A Regulatory Genes in Phaseolus vulgaris L.

**Figure 1:**
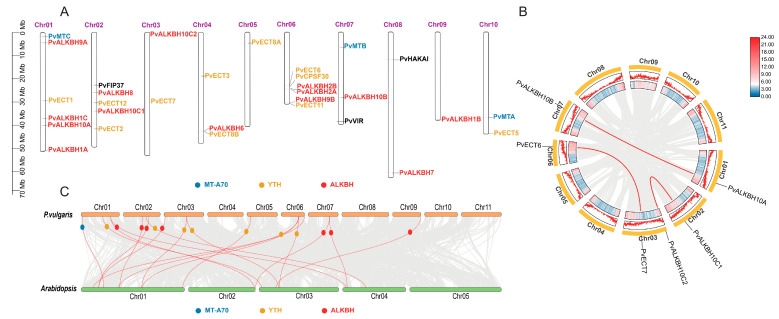
Chromosomal distribution and collinearity analysis of m^6^A regulatory genes in common bean. (**A**) Chromosomal localization of the 31 m^6^A regulatory genes. The scale on the left side of the figure was denoted in Mb. Each chromosome was labeled with its corresponding number at the top. (**B**) Synteny analysis of m^6^A regulatory genes in common bean. The outer side of the ring displays the chromosomal numbers, while the inside of the ring represented gene density in both heat map and line formats. Gray lines indicated all collinear blocks in the common bean genome, whereas red lines signified collinear relationships of m^6^A regulatory genes. (**C**) Interspecies synteny analysis between m^6^A regulatory genes in common bean and those in *Arabidopsis*. Gray lines represented all collinearity results, and red lines indicated homologous gene pairs.

A total of 31 *P. vulgaris* genes associated with the m^6^A system of *Arabidopsis* were identified, which were distributed across ten of the eleven chromosomes. The majority of these genes were located at the ends of the chromosomes, whether proximal or distal (Figure 1A). Notably, *PvALKBH2A* and *PvALKBH2B* were found in close proximity on chromosome 06, although no tandem duplication event was observed. A comprehensive genome-wide synteny analysis was conducted to explore how genome duplication events have contributed to the expansion of these gene families in the common bean. Three gene pairs were identified to be originated from segmental or whole genome duplication (WGD) events (Figure 1B), indicating that segmental duplication/WGD were possibly responsible for the expansion of the m^6^A regulatory gene family in the common bean. We calculated the Ka/Ks ratio, and the results indicate that all observed pairs were subjected to purifying selection. It is estimated that the original divergence between *PvALKBH10A* and *PvALKBH10B* occurred roughly 28.42 million years ago (Table 2). Additionally, a synteny analysis was carried out between *P. vulgaris* and *Arabidopsis*. This study identified eighteen pairs of orthologous genes, including 14 genes from svcommon bean and 13 from *Arabidopsis* (Figure 1C).

### 2.3. Evolutionary and Functional Key Residue Analyses of m^6^A Regulatory Gene Family in Phaseolus vulgaris L.

**Figure 2:**
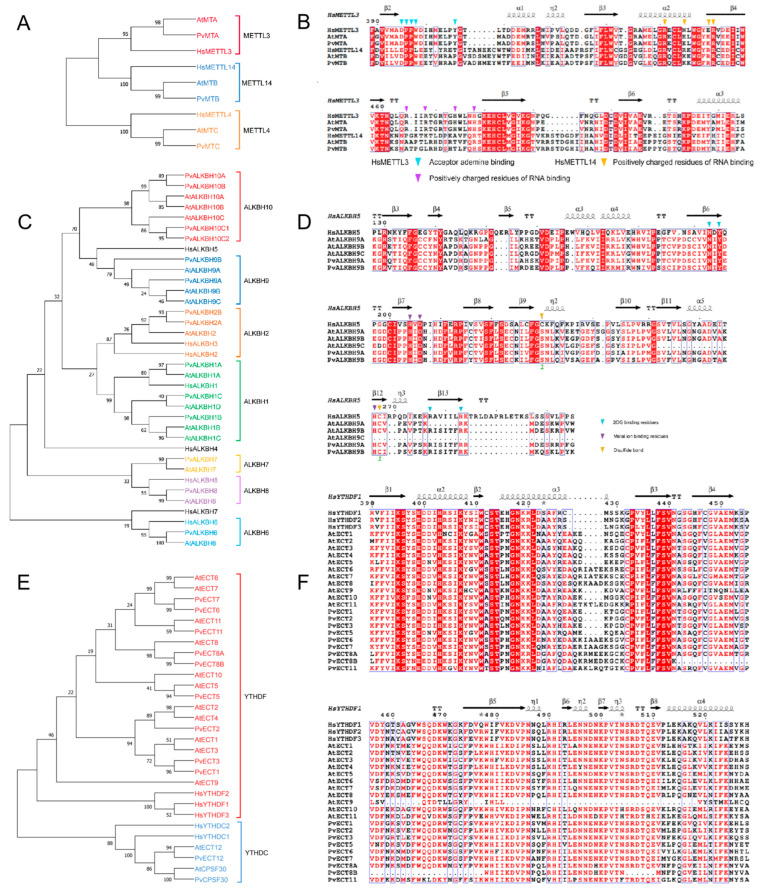
Phylogenetic and functional analyses of key residues in m^6^A writer/eraser/reader genes from common bean, *Arabidopsis*, and human: (**A**) phylogenetic tree of MT-A70 family proteins; (**B**) sequence alignment of METTL3 and METTL14 subclade proteins; (**C**) phylogenetic tree of ALKBH family proteins; (**D**) sequence alignment of ALKBH9 subclade proteins from common bean and *Arabidopsis*, as well as human HsALKBH5; (**E**) phylogenetic tree of YTH family proteins; (**F**) sequence alignment of YTHDF subclade proteins.

Our analysis revealed that the MT-A70 proteins from *P. vulgaris* were grouped into three distinct clades: METTL3 subclade (PvMTA), METTL14 subclade (PvMTB), and METTL4 subclade (PvMTC) (Figure 2A). The analysis of multiple sequence alignments indicated that several key functional sites are conserved, particularly the residues vital for interactions with AdoMet and for binding RNA (Figure 2B).

The proteins from the ALKBH family in *P. vulgaris* were grouped into seven subclades (Figure 2C). A comparative analysis of amino acid sequences of HsALKBH5 and ALKBH9 members revealed the conservation of several functional areas, featuring critical residues that play an important role in the binding of 2OG and metal ions. (Figure 2D).

In common bean, YTH proteins were divided into the YTHDF and YTHDC subclades, with the latter further categorized into two distinct subclades: PvYTHDC and PvCPSF30 (Figure 2E). Similar to *Arabidopsis*, the common bean features two YTH proteins classified within the YTHDC subclade. The YTHDF subclade had fewer proteins, with common bean containing nine, compared to eleven in *Arabidopsis.* Additionally, two copies of AtECT8 orthologs were identified in the common bean, which suggests potential functional diversity or redundancy. The alignment of multiple sequences from the YTHDF and YTHDC subclades revealed the conservation of various functional sites, including residues associated with the aromatic cage, m^6^A interaction, and RNA binding (Figure 2F).

Overall, these results suggest that m^6^A regulatory machinery was evolutionarily conserved between *Arabidopsis* and bean based on the conservation of residues on various functional sites.

### 2.4. Structural Characterization of m^6^A Regulatory Genes in Phaseolus vulgaris L.

**Figure 3:**
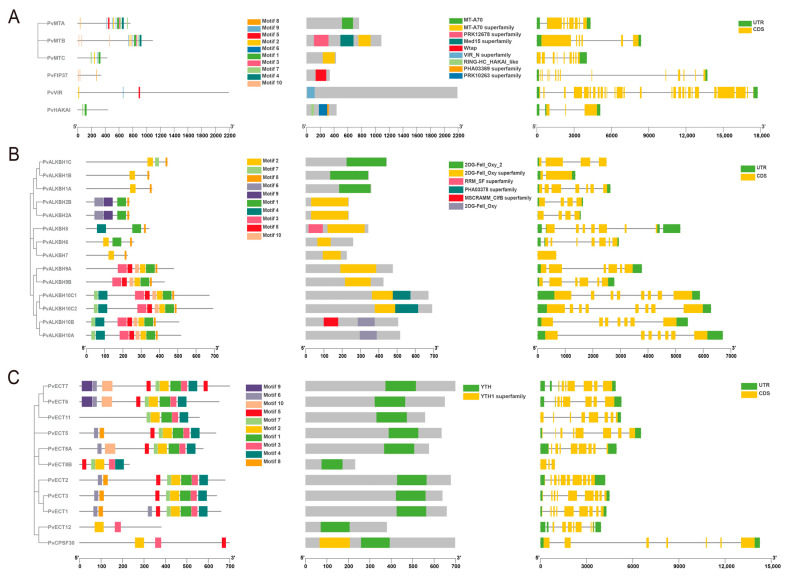
The phylogenetic relationships, conserved motifs, functional domains, and exon–intron organizations of m^6^A regulatory genes in common bean: (**A**) structure analysis of m^6^A writer genes; (**B**) structure analysis of m^6^A eraser genes; (**C**) structure analysis of m^6^A reader genes.

In the motif conservation analysis, it was observed that MTC lacked several motifs in comparison to PvMTA and PvMTB (Figure 3A). Furthermore, in addition to the MT-A70 protein domain, PvMTB featured two supplementary domains (Figure 3A). This observation suggests that PvMTB could be involved in various regulatory mechanisms. By utilizing TBtools (v1.098768), an exon-intron diagram for MT-A70 family was constructed, providing a comprehensive view of CDS, as well as the introns and untranslated segments. Genes belonging to the MT-A70 family usually have between 5 and 8 introns.

In the motif conservation analysis of ALKBH genes in *P. vulgaris*, it was observed that the motifs 1–3, 5, 8, and motif 10 were arranged similarly in both the PvALKBH9 and PvALKBH10 subclades. Notably, within the PvALKBH10 subclade, two additional motifs, namely, 4 and 7, were observed. Two specific motifs, 6 and 9, were used to define the PvALKBH2 subclade. PvALKBH8 contained three motifs: 1, 4, and 8. In comparison to PvALKBH7, PvALKBH6 displayed three motifs, along with an additional motif (Figure 3B). All PvALKBH proteins retained a conserved domain associated with either the 2OG-Fe(II)_Oxy, 2OG-Fe(II)_Oxy2, or 2OG-Fe(II)_Oxy superfamily (Figure 3B). The gene structures of the *PvALKBHs* showed significant diversity regarding the number and location of exons and introns, with the ALKBH family genes identified as having 1 to 8 introns (Figure 3B).

The majority of proteins belonging to the PvYTHDF subclade showed consistent motifs 1 to 7. Nonetheless, PvECT11 did not have motifs 5 and 6, and PvECT8B was deficient in motifs 1 and 6. Several members contained one or two extra motifs. PvYTHDC proteins consistently showed conserved motifs 2 and 3, and an additional motif 5 was present for PvCPSF30. These observations indicate a high level of conservation among YTH family proteins in common bean, with some minor evolutionary variations within subclades (Figure 3C). All PvYTHs contain a conserved domain (CDD: pfam04146) of comparable length that is characteristic of YTH proteins. It is worth mentioning that PvCPSF30 also featured the YTH1 superfamily domain (CDD: COG5084). Genes within the PvYTH cluster that belonged to the same clade displayed analogous gene structures, including similar counts and locations of exons and introns (Figure 3C).

Overall, these observations suggest that gene structure in m^6^A regulatory machinery was largely conserved, however, sequence variations existed among the members of MT-A70, ALKBH, and YTH. Such variations should be further characterized and functionally validated.

### 2.5. Detection of cis-Acting Elements in the Promoter Region of m^6^A Regulatory Genes in Phaseolus vulgaris L.

Among m^6^A regulatory genes, those that react to light, phytohormones, and stress were found to be more abundant compared to those associated with plant development and growth.

**Figure 4:**
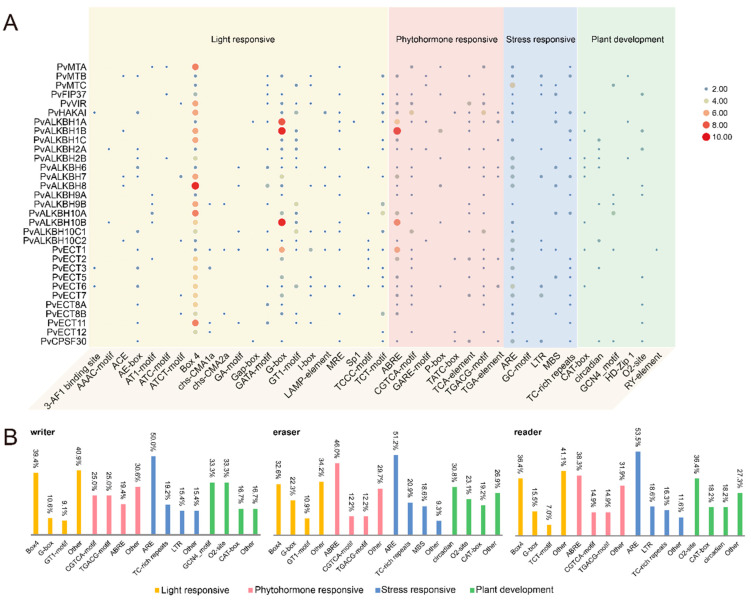
Localization and analysis of *cis*-acting elements in the promoter regions of m^6^A regulatory genes: (**A**) distribution of *cis*-elements in the promoter regions of m^6^A regulatory genes, with the size of the circles indicating the number of *cis*-elements present; (**B**) bar graphs representing the percentage of *cis*-acting elements in categories such as Light responsive, Phytohormone responsive, Stress responsive and Plant development among m^6^A writers, erasers and readers.

In the category of light responsiveness, all members of the target families contained the Box 4 element, except for PvCPSF30. The highest proportions recorded were 39.4%, 32.6%, and 36.4%. Among the *cis*-elements in the phytohormone group, ABRE was the most widespread, indicating a connection to abscisic acid responsiveness. It was succeeded by the CGTCA and TGACG motifs, which are related to the response to MeJA. We found several elements that respond to stress within the promoter regions of m^6^A regulatory genes, with ARE being the most prevalent in reaction to anaerobic induction (Figure S1). The promoter region of PvCPSF30 contained the GC motif, which is specifically linked to anoxic inducibility. cis-Elements associated with various aspects of plant growth and development were identified (Figure 4A,B). These components are connected to meristem expression, the control of circadian rhythms, endosperm function, the differentiation of palisade mesophyll, regulation of zein metabolism, and regulatory processes in seeds, respectively. It is noteworthy that a higher number of elements related to plant growth and development were found within the promoter area of ALKBH members compared to other m^6^A regulatory genes.

### 2.6. Alterations in the Expression of m^6^A Regulatory Genes in Common Bean Plants Infected with BCMV

**Figure 5:**
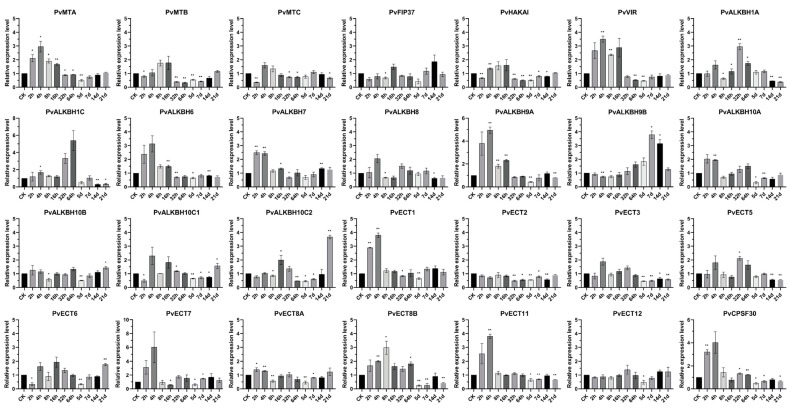
RT-qPCR analysis of m^6^A regulatory genes after viral infection. Tissue samples were collected at ten different time points post-BCMV treatment in equal amounts. Each value represents the mean ± SEM of three independent experiments. * Refers to significant differences at *p* < 0.05 and ** denotes significant differences with *p* < 0.01, compared to the control.

This research used BCMV as a biotic stressor to analyze changes in the expression of m^6^A regulatory genes in both healthy common bean plants and those exposed to the virus at various time points: 0, 2, 4, 8, 16, 32, and 64 h, along with 5, 7, 14, and 21 days following viral inoculation (Figure 5).

The transcript levels of components of the m^6^A writer complex, including *PvMTA*, *PvMTB*, *PvVIR*, and *PvHAKAI*, displayed similar patterns under the tested conditions. In virus-infected plants, these genes were mainly upregulated relative to controls within the first 16 h but then downregulated at 64 h and 5 days post-infection. Among the ALKBH family, most members, except for *PvALKBH9B* and *PvALKBH10C1/C2*, exhibited increased expression levels shortly after the onset of the viral infection, particularly between 2 and 4 h. *PvALKBH1* showed significant upregulation at 32 and 64 h, followed by downregulation at 14 and 21 days. Following viral infection, *PvALKBH9A* expression experienced a noticeable rise for one day, after which it returned to a pattern similar to the control. The expression of *PvALKBH10A* and *PvALKBH10B* fluctuated, displaying similar patterns after viral infection. At the onset of BCMV infection, the levels of *PvALKBH10C1/C2* increased, followed by a general decline, yet they remained notably elevated at the 21-day mark. *PvALKBH9B* displayed a different expression trend compared to other genes, starting with a slight downregulation at 8 h after inoculation, then showing a consistent rise that peaked at 7 days post-infection (dpi).

All the reader gene identified, with the exception of *PvECT5*, showed decreased levels at the 5-day mark. *PvECT1* showed a notable increase in expression during the first four hours following viral inoculation and remained relatively stable thereafter. *PvECT2* displayed a unique expression profile, continuously declining at every measured time interval. The expression levels of *PvECT3/5/8B* exhibited minor variations during the initial phases of viral infection but generally decreased after five days. Two hours after infection, *PvECT6* was notably downregulated, whereas at 21 days, it exhibited a significant upregulation. After the viral infection, the expression of *PvECT11* and *PvCPSF30* was found to rise within four hours but then later declined.

### 2.7. PvMTA Interacts with PvMTB in Phaseolus vulgaris L.

**Figure 6:**
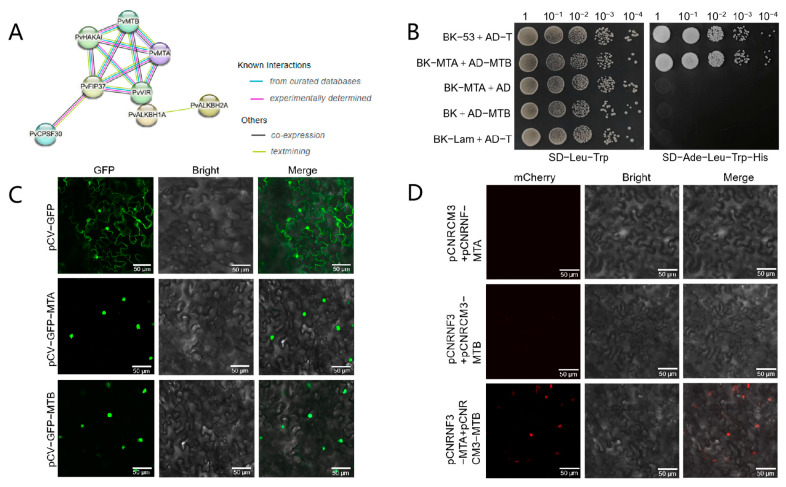
PvMTA interacts with PvMTB in *Phaseolus vulgaris* L. (**A**) The protein–protein interaction network of 31 m^6^A regulatory genes in common bean constructed with STRING v11.5 (interaction scores > 0.900). The network nodes represent proteins and edges represent protein–protein associations. Different colors indicate various types of interactions as specified in the legend. (**B**) Interactions between MTA and MTB as demonstrated by the Y2H assay. The transformants were cultured on SD/-Leu/-Trp (-LW) and subsequently selected on SD/-Leu/-Trp/-His/-Ade (-LWHA). (**C**) Subcellular localization of MTA and MTB. Scale bar = 10 μm. (**D**) Interactions between MTA and MTB assessed by BiFC assay. MTA and MTB were fused to the N- and C-terminal fragments of red fluorescent protein (mCherry). The MTA-MTB interaction led to the reconstitution of a fluorescence-competent structure and the restoration of red fluorescence. Scale bar = 20 μm.

Our study involved the development of interaction networks featuring 31 m^6^A regulatory proteins. The alignment of these proteins with thirty STRING proteins helped us to outline their connections. It is important to highlight that all m^6^A writer components, except for PvMTC, showed significant interactions. This observation suggested that these elements could have pivotal functions in m^6^A modification through the establishment of protein complexes (Figure 6A).

Interactions between PvMTA and PvMTB were examined using the Y2H system. Yeast cells that co-expressed MTA and MTB showed typical growth on selective media, indicating interaction between PvMTA and PvMTB (Figure 6B). By transiently expressing MTA-GFP and MTB-GFP fusion proteins in the leaves of *N. benthamiana*, we conducted a subcellular localization analysis that demonstrated that both proteins are present in the nucleus (Figure 6C). BiFC (Bimolecular Fluorescence Complementation) assays were also utilized to examine the interaction between PvMTA and PvMTB. PvMTA was attached to the N-terminal portion, whereas PvMTB was connected to the C-terminal portion of mCherry to facilitate further analysis. The assays demonstrated that the interaction of PvMTA and PvMTB within the nucleus resulted in the reassembly of the fluorescence-competent structure, leading to the emission of red fluorescence (mCherry) (Figure 6D).

### 2.8. Characterizations of m^6^A Methyltransferases in Phaseolus vulgaris L.

**Figure 7:**
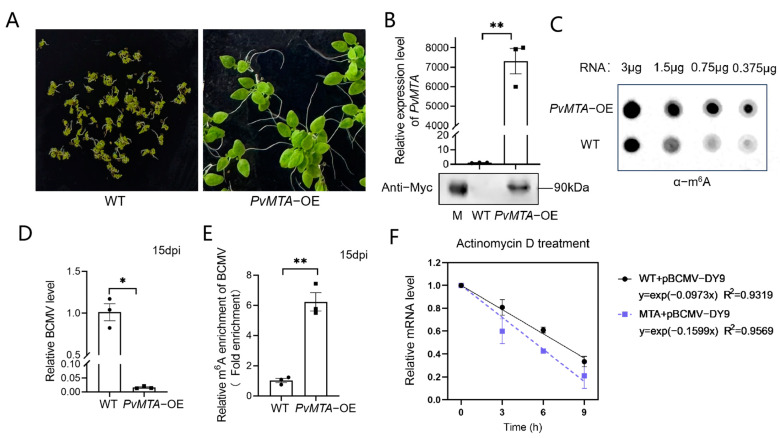
Overexpression of PvMTA negatively regulates BCMV infection through mechanisms mediated by m^6^A modification. (**A**) Generation of transgenic *N. benthamiana* plants overexpressing *PvMTA* gene. The left side of the plate shows the growth of wild type, which exhibited abnormal growth in hygromycin-elective medium, and the right side shows that the mutants exhibited the hygromycin resistance phenotype. (**B**) RT-qPCR and WB analyses indicate that *PvMTA* was effectively translated and expressed in the mutants. The values were presented as the mean ± SEM (two-sided *t*-test, n = 3, ** *p* < 0.01) (**C**) The overall levels of m^6^A modification of the wild-type and *PvMTA*-OE plants were investigated by dot blotting assay. (**D**) The accumulation of BCMV RNA in the BCMV-infected transgenic and wild-type plants was assessed using RT-qPCR with CP gene-specific primers. The values are presented as the means ± SEM (two-sided *t*-test, n = 3, * *p* < 0.05). (**E**) m^6^A-IP-qPCR assay was performed to detect the m^6^A variation in BCMV gRNA in BCMV-infected wild-type/*PvMTA-OE* plants. The values are expressed as the mean ± SEM (two-sided *t*-test, n = 3, ** *p* < 0.01). (**F**) The gRNA stability of BCMV was evaluated, with TI representing transcription inhibition. The values were presented as means ± SD for three biological replicates.

To preliminarily verify the methyltransferase ability of PvMTA and examine how the identified gene contributes to the disease resistance in common bean plants, we overexpressed this gene using the 35S cauliflower mosaic virus promoter in *N. benthamiana*. After screening in a subculture medium with 20 mg/L hygromycin, we successfully obtained three stable T3 generation transgenic lines of *N. benthamiana* (Figure 7A).

The results from both the RT-qPCR and Western blot indicate that these homozygous mutants successfully exhibited higher levels of *PvMTA* expression in comparison to the wild-type plants (Figure 7B). The transgenic line with highest expression level was selected for subsequent analysis. The m^6^A dot blotting analysis demonstrated that *PvMTA* underwent successful translational expression in the mutant plants and consequently, there was a rise in the total m^6^A levels relative to the wild type, which further validates the m^6^A methylase activity of PvMTA (Figure 7C).

### 2.9. Overexpression of PvMTA Negatively Regulates BCMV Infection Through Mechanisms Mediated by m^6^A Modification

To further elucidate the function of *PvMTA* during BCMV infection, T3 generation transgenic plants expressing *PvMTA*-OE were subjected to BCMV inoculation. Subsequently, the plants were analyzed for BCMV RNA accumulation at 2 weeks post-inoculation (wpi) through RT-qPCR. Remarkably, the accumulation of BCMV RNA was notably reduced in *PvMTA-OE* lines in comparison to wild-type (*N. benthamiana*) plants (Figure 7D). This finding suggests that *PvMTA* may act as a host factor that exerts a negative regulatory effect on virus infection.

To investigate whether *PvMTA* influences the extent of m^6^A modifications on BCMV RNAs, total RNA was extracted from BCMV-infected *PvMTA-OE* mutant plants and their wild-type counterparts. An RNA immunoprecipitation assay (RIP) utilizing the anti-m^6^A antibody was then conducted to isolate m^6^A-modified RNAs. The m^6^A content of BCMV was subsequently quantified through m^6^A-IP-qPCR in both BCMV-infected *PvMTA-OE* mutant plants and WT controls. Interestingly, the mutant plants exhibited significantly higher m^6^A levels in BCMV than the wild-type plants (Figure 7E).

Transcription inhibition assays employing actinomycin D were executed to gauge the half-life of BCMV genomic RNA in BCMV-infected *PvMTA-OE* mutant plants and their wild-type equivalents, and the data revealed that BCMV genomic RNA underwent accelerated degradation in BCMV-infected *PvMTA-OE* mutant plants when contrasted with the wild-type plants (Figure 7F).

### 2.10. Silencing of PvMTA Promotes BCMV Infection Through Mechanisms Mediated by m^6^A Modification

**Figure 8:**
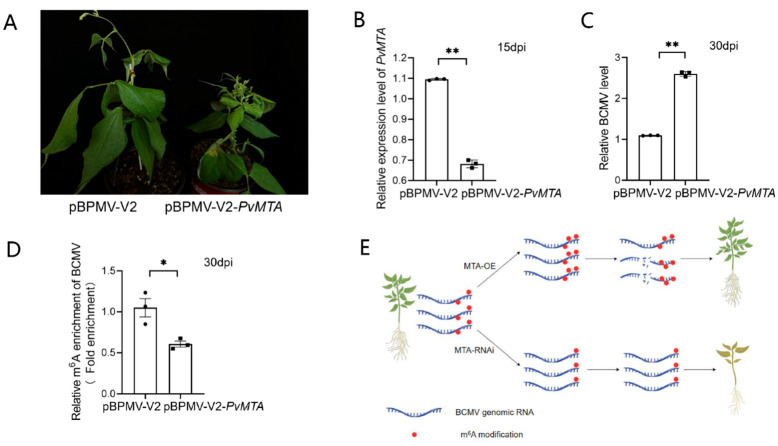
Silencing of PvMTA promotes BCMV infection through mechanisms mediated by m^6^A modification. (**A**) BCMV-induced symptoms in beans with silenced *MTA* and those expressing normal levels of *MTA*. (**B**) RT-qPCR analyses indicate that *PvMTA* was effectively silenced through BPMV-mediated VIGS vector. The values were presented as the mean ± SEM (two-sided *t*-test, n = 3, ** *p* < 0.01). (**C**) The accumulation of BCMV gRNA in the *MTA*-silenced and control bean plants was assessed using qPCR with CP gene-specific primers. The values are presented as means ± SEM (two-sided *t*-test, n = 3, ** *p* < 0.01). (**D**) m^6^A-IP-qPCR assay was performed to detect m^6^A variation of BCMV gRNA in BCMV-infected *MTA*-silenced and control bean plants. The values are expressed as the mean ± SEM (two-sided *t*-test, n = 3, * *p* < 0.05). (**E**) Schematic representation of the potential molecular mechanism underlying *MTA* gene regulation of viral infection in common bean.

To further explore the impact of *PvMTA* on BCMV infection, we used BPMV vectors to induce *PvMTA* silencing in *Phaseolus vulgaris* L. The results presented in Figure 8B showed a decrease in *PvMTA* expression in plants treated with pBPMV-V2-PvMTA compared to control (pBPMV-V2). The silencing of *PvMTA* did not alter the growth phenotypes of the plants. Subsequently, plants silenced for *PvMTA*, along with those pre-treated with pBPMV-V2, were exposed to BCMV. Plant beans *PvMTA*-silenced showed more severe symptoms than those expressing normal levels of *PvMTA* (Figure 8A). In contrast to the control group, plants with silenced *PvMTA* exhibited elevated levels of BCMV RNA accumulation (Figure 8C). To further probe this phenomenon, m^6^A-IP-qPCR assays were performed to assess the m^6^A content of BCMV in plants infected with BCMV and pre-treated with either pBPMV-V2-PvMTA or pBPMV-V2. The findings indicate a reduced m^6^A level of BCMV in plants pre-treated with pBPMV-V2-PvMTA compared to those pre-treated with pBPMV-V2 (Figure 8D).

## 3. Discussion

### 3.1. Features of the m^6^A Regulatory Gene Family

Our study conducted a detailed and methodical genome-wide examination of m^6^A regulatory genes in *P. vulgaris*. We identified 31 genes which we organized into three groups. Previous studies have highlighted the significance of both structural similarities and diversities in the evolution of gene families [40,41,42,43,44], and we found that the expansion of m^6^A regulatory genes in *P. vulgaris* is largely a result of segmental and whole-genome duplication.

The presence of *cis*-acting elements in the promoter regions suggests that m^6^A can be triggered by diverse molecular switches and that may play a significant role in regulating a broad network of genes associated with light response, phytohormones, stress, and developmental processes. The examination of PPI predicted that the components of the “writer complex” components may interact with each other, with a high confidence level of over 0.900 in the network. These observations are in agreement with prior research in *Arabidopsis* [45]. As a result, we analyzed the expression patterns of multiple identified genes in *P. vulgaris* following BCMV infection. The complex and diverse gene expression patterns related to m^6^A modification in common beans may be linked to their functions during this viral infection [6,46].

### 3.2. Roles of PvMTA in the Context of BCMV Infection in Common Bean

Earlier research has shown that the m^6^A modification plays a role in how plants respond to different biotic stresses [6,7,8,37,38,47,48], and the RT-qPCR findings indicate that a majority of the m^6^A regulatory genes responded to BCMV infection in common bean, suggesting their potential involvement in regulating the virus–host interaction [6,7,37,38]. Among these genes, *PvMTA*, identified as the core component of the methyltransferase complex, was selected for functional validation. Notably, only a single copy of *MTA*, *MTB*, and *MTC* was identified in *P. vulgaris*. We hypothesized that the identified *PvMTA* likely serves as the catalytically active component of the m^6^A methyltransferase complex in beans. Since bean plants lack of an effective transformation system, the methyltransferase activity of PvMTA was investigated through the construction and screening of *PvMTA* overexpression *N. benthamiana* mutants as well as through the silencing of *PvMTA* using the BPMV-mediated vector [49,50,51]. Results indicated that plants with elevated *PvMTA* expression exhibited a greater overall level of m^6^A modification than wild type, while silencing the gene led to a reduction in these levels, highlighting the m^6^A methyltransferase activity of PvMTA. In mammals, METTL3 forms a heterodimer with METTL14 in a 1:1 ratio through the methyltransfer structural domain MTD, which enhances the catalytic activity of METTL3 [52,53]. MTA, as a core component of the methyltransferase complex in plants, is the direct homolog of METTL3, whereas MTB corresponds to the immediate homolog of METTL14 in plants [29]. The heterodimerization of MTA with MTB has been confirmed in *Arabidopsis* [45], apple [54], and strawberry [55]. The protein interaction network predictions suggest a potential interaction between *PvMTA* and *PvMTB*. Consequently, the present study focused on determining the subcellular localization of PvMTA and PvMTB and to explore their interaction through Y2H and BiFC methods. Collectively, the findings suggested that PvMTA and PvMTB in common bean are localized in the nucleus and directly interact. This supports the notion that PvMTA functions as an m^6^A methyltransferase and likely interacts with PvMTB in the nucleus to carry out relevant biological functions.

The MTA protein is deeply involved in overseeing the mechanisms that dictate how plants grow and develop. Studies indicate that MTA is vital for the proper embryonic development of *Arabidopsis* [27]. Compared to wild-type, *mta Arabidopsis* mutants with decrease m^6^A abundance exhibit various phenotypic changes including reduced apical dominance, deformed floral organs, increased trichome branching, shortened root growth, and defects in primary xylem and gravitropism development [28,29,56].

Compared to the wild type, rice plants with mta2 and MTA2 mutations demonstrate shorter panicles, lower seed setting rates, and fewer effective grains [57]. Both mta2 and MTA2 mutants in rice exhibit shorter spike lengths than the wild type, which leads to a decrease in yield and the number of viable seeds [57]. MTA plays a role in strawberries by modulating the expression of crucial genes involved in ABA synthesis and signaling, such as NCED5, ABAR, and AREB1. This modulation occurs through changes in their m^6^A abundance, ultimately affecting the maturation process of strawberry fruit [55]. In addition, MTA significantly contributes to how plants manage stress. For instance, under drought conditions, the m^6^A modification facilitated by MTA boosts the drought resistance of apple trees by stabilizing mRNA and improving the translation efficiency of genes linked to oxidative stress and lignin accumulation [54]. In *Arabidopsis*, *MTA* expression is notably upregulated under salt stress, and lines with *ABI3*:*MTA* and *MTA* knockdown exhibit salt-sensitive characteristics in a manner reliant on m^6^A [10]. While the involvement of m^6^A in regulating pathogen infection has been reported, the investigation into the genetic function of *MTA* in response to biotic stress is still in its nascent phase. Studies have shown that the overexpression of *MTA* and *HAKAI* in tomatoes and *N. benthamiana* [37,38] leads to an elevation in m^6^A levels on PepMV, as a result, the accumulation of PepMV RNA is suppressed. Conversely, silencing of *MTA* and *HAKAI* decreases m^6^A levels on PepMV RNA, thereby facilitating viral infection.

The impact of PvMTA-mediated m^6^A modification on the stability of the BCMV genome in *N. benthamiana* was investigated through the heterologous overexpression of *PvMTA*. Actinomycin D was utilized to halt plant transcription, allowing for the examination of the relationship between m^6^A abundance and the stability of the viral genomic RNA [7]. Our findings revealed that m^6^A modification by *PvMTA* resulted in an increase in the m^6^A levels of the BCMV genome, leading to decreased genomic RNA stability and reduced viral accumulation. This suggests a negative correlation between PvMTA-mediated m^6^A modification and BCMV genomic RNA stability. Notably, no significant phenotypic changes were observed in either experimental condition, with the reasons for this remaining to be determined. A study conducted by He et al. [38] further indicates that the regulation of PepMV infection via m^6^A modification primarily involves downstream NbECT2 recognition of m^6^A, which triggers the recruitment of NMD-related factors that degrade viral RNA, thereby hindering viral infection. In contrast, PepMV-encoded RdRP was found to interact with SlHAKAI protein and autophagy-related protein SlBeclin1 [37]. The recruitment of SlBeclin1 by PepMV RdRP promoted the autophagic degradation of SlHAKAI protein through the autophagy pathway, inhibiting the plant defense response mediated by m^6^A modification. Whether a similar molecular mechanism underlies the regulatory role of *MTA*-mediated m^6^A modification on BCMV infection in beans remains to be further verified.

## 4. Materials and Methods

### 4.1. Identification and Characterization of m^6^A Regulatory Genes in Phaseolus vulgaris L.

In this study, the *Phaseolus vulgaris* v2.1 genome was obtained from Phytozome v13 (http://phytozome.jgi.doe.gov/, accessed on 20 June 2022). To identify bean m^6^A regulatory genes, we utilized the amino acid sequences of *Arabidopsis* m^6^A-related proteins and queried the common bean genome through BLASTP with the standard parameters in TBtools (v1.098768) [58].

To evaluate the predicted sequences of m^6^A regulatory genes in *P. vulgaris* L., we employed the hidden Markov model (HMM) profiles from the MT-A70 family (PF05063), 2OG-Fe(II) oxygenase superfamily (PF13532), YTH domain (PF04146), WTAP family (PF17098), and virilizer domain (PF15912) [58]. These sequences were additionally confirmed through the use of the Conserved Domain Database [59]. Furthermore, the peptide sequences’ physicochemical properties, such as molecular weight (MW) and isoelectric points (pI), were analyzed with the help of ExPASy (https://web.expasy.org/compute_pi/, accessed on 20 June 2022) [60].

### 4.2. Chromosome Location, Collinearity Relationships, Gene Duplication Events, and Evolutionary Selection

Details about the locations of m^6^A regulatory genes and their corresponding gene family members were extracted from the GFF3 files of the bean genome and visualized using the TBtools software. Duplication events among m^6^A regulatory genes were examined using MCScanX [61]. Collinearity analysis was conducted using TBtools. To assess the evolutionary selection pressure influencing m^6^A regulatory genes, we calculated the ratio of Ka to Ks substitution rates between pairs of genes using TBtools. As noted in earlier findings, the divergence time for the duplicated gene pairs has been estimated [62].

### 4.3. Phylogeny, Conserved Motifs, Domains, Gene Structure, cis-Elements, and the Protein–Protein Interactions Analyses

Full-length amino acid sequences (Supplementary Table S2) were aligned using MEGA10.1.7 [63]. A maximum likelihood (ML) tree was constructed with partial deletion, poison correction, and 1000 bootstrap replicates. The alignment’s secondary structure was annotated using ESPript (https://espript.ibcp.fr, accessed on 21 June 2022) [64]. We acquired the secondary structures of the human m^6^A regulatory gene family from the NCBI structure database [65,66,67].

After analyzing the conserved motifs with MEME Suite (https://meme-suite.org/meme/, accessed on 21 June 2022) [68], the resulting file was downloaded. Using the Batch CD-Search tool (https://www.ncbi.nlm.nih.gov/cdd/, accessed on 22 June 2022), we examined the conserved domains of multiple full-length protein sequences and subsequently downloaded the output file. Gene lengths, structures, phylogenetic tree, and bean GFF file were used in TBtools for integrated visualization.

*Cis*-regulatory elements (CREs) were identified from the promoter sequence extending 2000 base pairs upstream of the initiation codon of the m^6^A regulatory genes [69] and visualized using TBtools. The STRING v11.5 database was utilized to construct protein–protein interaction (PPI) networks [70]. Thirty-one m^6^A regulatory proteins were uploaded, resulting in the identification of 30 interacting STRING proteins with interaction scores above 0.900, signifying the highest confidence level.

The promoter sequence extending 2000 base pairs upstream of the initiation codon was obtained from the FASTA file of the common bean genome using TBtools.

### 4.4. Plant Materials, Inoculation, and RT-qPCR Analysis

The experimental plants of the bean variety Stringless Green Refugee (SGR) were cultivated in a growth chamber with a 1 h day at 26 °C and an 8 h night at 20 °C. The plants were inoculated using a mechanical method once the first set of true leaves had fully developed, usually around the seventh day. The BCMV strain DY9 used in this study was sourced from Jinzhong, Shanxi, and maintained in the laboratory. The treatment and control (PBS) groups were then transferred to a greenhouse under the same conditions as the climate chamber for further cultivation.

Leaves from both virus-infected and mock-treated plants were sampled at 0, 2, 4, 8, 16, 32, and 64 h, and at 5-, 7-, 14-, and 21-days post-inoculation for RT-qPCR [28,39]. For RT-qPCR analysis, total RNA was extracted from common bean samples using Trizol reagent (Biosharp, Beijing, China), followed by first-strand cDNA synthesis using RT SuperMix for qPCR kit (Vazyme, Nanjing, China). The RT-qPCR reactions were carried out using gene-specific primers (see Supplementary Table S3), following the amplification parameters outlined in previous reports [39]. The relative expression levels of the genes were assessed using the 2^−ΔΔCT^ method [71] and presented as the mean ± SEM.

### 4.5. Y2H Analysis

A Y2H analysis was conducted following established procedures [55]. Briefly, the coding sequences of *PvMTA* and *PvMTB* were extracted from bean cDNAs, amplified, and then introduced into the pGBKT7 (BK) and pGADT7 (AD) vectors. The primers used were given in Supplementary Table S4. The *Saccharomyces cerevisiae* strain Y2H Gold was co-transformed with these plasmids. Initially, the yeast cells were cultured in SD/-Leu-Trp (-LW) (Huayueyang, Beijing, China) before being transferred to SD/-Leu-Trp-His-Ade (-LWHA) medium for additional growth.

### 4.6. BiFC and Subcellular Localization

The coding sequences (CDS) for bean MTA and MTB, with their stop codons eliminated, were amplified from cDNAs and then inserted into the PCV-GFP-N2 vector. A bimolecular fluorescence complementation (BiFC) assay was employed to evaluate the MTA-MTB interaction. The N-terminal and C-terminal fragments of mCherry were linked to MTA and MTB, respectively. The CDS of MTA and MTB, excluding stop codons, were amplified by PCR (Supplementary Table S4) and inserted into the pCNRNF3 and pCNRCM3 vectors, resulting in the creation of the pCNRNF3-MTA and pCNRCM3-MTB constructs. The constructs generated were transformed into GV3101, which was subsequently used to infiltrate tobacco leaves to enable the individual expression of N-mCherry-MTA and C-mCherry-MTB, as well as the simultaneous expression of both fusion proteins. We transformed the vectors p35S::MTA-GFP and p35S::MTB-GFP into *Agrobacterium tumefaciens* and then infiltrated the *Nicotiana benthamiana* leaves with them using a syringe to achieve transient expression. After a culture period of 48 h, we used a confocal microscope (Olympus FV3000, Olympus, Tokyo, Japan) to examine the region that had been infiltrated.

### 4.7. Generation of Transgenic N. benthamiana Plants Overexpressing PvMTA

A full-length *PvMTA* was cloned using the *PvMTA*-F and *PvMTA*-R primers (Supplementary Table S4) and subsequently inserted into the binary vector pBWA(V)HS. The vector produced was transformed into *A. tumefaciens* EHA105 cells(WEIDI, Shanghai, China), which were subsequently employed to transform *N. benthamiana* through the leaf disc infection technique [49]. The regenerated plantlets were assessed using a subculture medium that contained 8 g/L agar, 4.4 g/L MS, and 20 mg/L hygromycin (Coolaber, Beijing, China). Two weeks later, the surviving seedlings from the selective medium were transferred to soil for seed production. Transgenic plants (*PvMTA*-OE) were confirmed using RT-qPCR and Western blot assays.

### 4.8. Western Blot Analysis

Proteins were obtained from the *PvMTA*-OE mutant plant using a lysis buffer composed of 4.5% (*w*/*v*) SDS, 50 mM Tris-HCl (pH 6.8), 7.5% (*v*/*v*) 2-mercaptoethanol, and 9 M urea (Coolaber, Beijing, China). After extraction, the proteins were separated through 10% SDS-PAGE, utilizing wild-type *N. benthamiana* as the negative control. Subsequently, the proteins were electrotransferred onto a nitrocellulose membrane (Boster, Wuhan, China). The membrane was incubated overnight at 4 °C in a solution of 5% non-fat milk in TBST buffer to block it effectively. We carried out immunoblotting by first incubating the membrane with an anti-Myc antibody (diluted 1:2500) (Sangon, Shanghai, China) for two hours, then performing a secondary incubation with the HRP-conjugated antibody (Sangon, Shanghai, China) at room temperature for an additional two hours. The ECL luminescence reagent (absin, Shanghai, China) was used to visualize the immunoreactive bands. All commercial antibodies used in this study were procured from Sangon Biotech (Shanghai, China) (https://www.sangon.com/ accessed on 22 June 2022).

### 4.9. m^6^A Dot Blot Assay

The m^6^A dot blot assay was carried out according to the protocol, with minor modifications as outlined in a previous study [37,38]. The RNA extraction was performed on samples from both the wild type (WT) and the *PvMTA* mutant and then subjected to denaturation by heating at 95 °C for 5 min to eliminate secondary structures. Subsequently, the denatured RNA was promptly cooled on ice and diluted stepwise to concentrations of 3000, 1500, 750, and 375 ng/μL with RNase-free water. Each diluted RNA sample (2 μL) was applied to a nitrocellulose membrane (Boster, Wuhan, China). The membrane underwent crosslinking at 37 °C for 30 min, after which it was washed with TBST for 5 min to eliminate any RNA that was not bound. Subsequently, the membrane was blocked and incubated with primary (m^6^A, SySy, Gottingen, Germany) and secondary antibodies (Sangon, Shanghai, China). Finally, the ECL luminescence reagent (absin, Shanghai, China) was utilized for the visualization of the immunoprecipitated spots.

### 4.10. m^6^A-IP-qPCR

The m^6^A-IP-quantitative PCR (qPCR) procedure was conducted following the methodology previously described in [72]. In brief, 150 μg of total RNA was extracted from mutant and wild-type *N. benthamiana* plants (*PvMTA-OE*) at 4 weeks post-inoculation. To remove any DNA contamination, total RNA was treated with DNase I (Beyotime, Shanghai, China) [73]. Subsequently, the RNA was purified by precipitation using glycogen (Beyotime, Shanghai, China, 25 μg/mL final concentration) and isopropanol. The resulting pellet was dissolved in ultra-pure H_2_O and the integrity of the RNA was assessed by electrophoresis on an agarose gel. A 10% (*V*/*V*) portion of the RNA was retained for the input sample, and the remaining RNA was evenly divided into two aliquots. One aliquot was incubated with 4 μg of anti-m^6^A polyclonal antibody (SySy, Gottingen, Germany), and the second one with 4 μg of anti-IgG antibody(Sangon, Shanghai, China) for 4 °C for 4 h in 500 μL of IP buffer [400 U of RNase Inhibitor (Thermo Scientific, Waltham, MA, USA), 150 mM NaCl, 10 mM Tris-HCl (pH 7.4), 0.1% (*v*/*v*) IGEPAL CA-630, and 2 mM RVC (Beyotime, Shanghai, China)]. Subsequently, 40 μL of Protein A/G magnetic beads was added to each sample (Thermo Scientific, Waltham, MA, USA) and incubated at 4 °C for at least 6 h. The RNA reaction mixture was extensively washed and used for RT-qPCR analysis.

### 4.11. RNA Stability Assay

Leaf samples from wild-type *N. benthamiana* and mutant (*PvMTA-OE*) plants infected with BCMV were trimmed into round discs (9 mm in diameter) and immersed in a solution of actinomycin D (Amyjet, Wuhan, China, 10 μg/mL). After a 1 h infiltration period, five discs were collected as controls for the initial time point. Subsequent samples were collected every three hours and the expression of *PvMTA* was analyzed by RT-qPCR (Supplementary Table S4) [74]. The relative expression of the gene at each time point, compared to the control, was used to calculate the mRNA degradation rate.

### 4.12. BPMV-Based Silencing of PvMTA

Bean pod mottle virus (BPMV) based vectors [50,51] were used to induce silencing of *PvMTA* in bean. A 300 bp fragment of *PvMTA* was introduced into the *BamH I* site of pBPMV-IA-V2 vector (see Supplementary Figure S2), while the empty vector lacking the target gene served as control. The pBPMV-IA-V2 is a DNA-based VIGS vector that could be directly inoculated onto bean plants. In brief, bean seedlings were treated in dark for 24 h before virus inoculation [50,75]. For the primary inoculation, a DNA-plasmid mixture containing equal amounts (5 μg) of pBPMV-IA-R1 and pBPMV-IA-V2 was prepared in PBS buffer. Afterward, for the secondary inoculations, SGR leaves infected with BPMV were blended in a mortar to produce leaf sap. The inoculation process involved mechanically rubbing the primary leaves according to the procedure established by Pflieger and colleagues [75]. Inoculated plants were then cultivated in a growth chamber set to a 16 h light and 8 h dark cycle. The silencing level of *PvMTA* was assessed by RT-qPCR. Silenced plants were then inoculated with BCMV and used for m^6^A-IP-qPCR analysis.

## 5. Conclusions

We characterized the m^6^A regulatory gene family in *P. vulgaris* and investigated how the expression of the identified m^6^A regulatory genes change in response to viral infection. *PvMTA* was isolated as a potential candidate gene that regulates BCMV infection in common bean. Functional validation of *PvMTA* revealed its m^6^A methylation transferase activity and demonstrated a reciprocal relationship between PvMTA and PvMTB. *PvMTA*, as a key m^6^A-associated component, showed responsiveness to BCMV infection. The gene function study of *PvMTA* was conducted using the overexpression mutant of this gene in *N. benthamiana* and plant materials after silencing *PvMTA* via the VIGS system. The results show that *PvMTA* positively regulated m^6^A abundance in BCMV genomic RNA and negatively regulated BCMV infection (Figure 8E). This study’s findings enhance our understanding of how m^6^A modification contributes to the antiviral response in common bean and provide new ideas for breeding disease resistance in common beans from an epigenetic perspective.

## Figures and Tables

**Table 1:** m^6^A genes detected in the *P. vulgaris* L. genome.

Gene Name	Locus ID	Group	Chr	Protein Property		
				Length (aa)	pI	MW (kDa)
PvMTA	Phvul.010G102500.1	MT-A70	10	761	6.01	84.32
PvMTB	Phvul.007G073300.1		7	1086	6.86	120.67
PvMTC	Phvul.001G016200.1		1	427	8.04	48.64
PvALKBH1A	Phvul.001G262100.1	ALKBH	1	358	6.11	40.36
PvALKBH1B	Phvul.009G262600.1		9	344	9.47	37.40
PvALKBH1C	Phvul.001G131400.1		1	443	8.89	49.26
PvALKBH2A	Phvul.006G137400.1		6	235	9.21	27.06
PvALKBH2B	Phvul.006G137611.1		6	235	9.21	27.02
PvALKBH6	Phvul.004G131600.1		4	259	5.79	29.74
PvALKBH7	Phvul.008G264300.1		8	224	6.36	25.44
PvALKBH8	Phvul.002G123600.1		2	342	7.59	38.29
PvALKBH9A	Phvul.001G044000.2		1	476	5.92	53.60
PvALKBH9B	Phvul.006G214800.1		6	425	8.83	48.48
PvALKBH10A	Phvul.001G147800.1		1	516	5.89	56.99
PvALKBH10B	Phvul.007G168900.1		7	505	6.44	55.72
PvALKBH10C1	Phvul.002G181800.1		2	671	6.36	73.15
PvALKBH10C2	Phvul.003G014200.1		3	691	6.18	74.54
PvECT1	Phvul.001G110200.1	YTH	1	658	7.96	72.22
PvECT2	Phvul.002G247000.1		2	677	6.02	74.03
PvECT3	Phvul.004G080300.1		4	638	5.96	70.15
PvECT5	Phvul.010G165400.1		10	634	5.25	69.57
PvECT6	Phvul.006G121600.1		6	649	5.84	71.55
PvECT7	Phvul.003G119300.1		3	698	7.59	77.24
PvECT8A	Phvul.005G045600.1		5	575	6.76	63.27
PvECT8B	Phvul.004G132700.1		4	231	9.34	26.62
PvECT11	Phvul.006G218800.1		6	557	9.51	62.58
PvECT12	Phvul.002G152600.1		2	379	5.38	42.68
PvCPSF30	Phvul.006G130200.1		6	697	6.28	76.46
PvFIP37	Phvul.002G107400.1		2	337	5.41	38.15
PvVIR	Phvul.007G267500.1		7	2188	5.35	240.79
PvHAKAI	Phvul.008G108800.1		8	436	6.16	47.61

**Table 2:** The evolutionary analysis of duplicated m^6^A regulatory genes and estimated divergence times.

Gene I	Location	Gene II	Location	Type of Duplication	K_a_	K_s_	K_a_/K_s_	T = K_s_/2r(MYA)
PvALKBH10A	1	PvALKBH10B	7	Segmental/WGD	0.126348392	0.852865956	0.148145662	28.42
PvECT6	6	PvECT7	3	Segmental/WGD	0.174929236	0.683184232	0.256049874	22.77
PvALKBH10C1	2	PvALKBH10C2	3	Segmental/WGD	0.189372849	0.669639206	0.28279833	22.32

## Data Availability

The original contributions presented in this study are included in the article/Supplementary Material. Further enquiries can be directed to the corresponding authors.

## Supplementary Materials

The following supporting information can be downloaded at: https://www.mdpi.com/article/10.3390/ijms26062748/s1.

## References

[1] Cortés A.J., Monserrate F.A., Ramírez-Villegas J., Madriñán S., Blair M.W. (2013). Drought tolerance in wild plant populations: The case of common beans (*Phaseolus vulgaris* L.). PLoS ONE.

[2] Worrall E.A., Wamonje F.O., Mukeshimana G., Harvey J.J., Carr J.P., Mitter N. (2015). Bean Common Mosaic Virus and Bean Common Mosaic Necrosis Virus: Relationships, Biology, and Prospects for Control. Adv. Virus Res..

[3] Flores-Estévez N., Acosta-Gallegos J.A., Silva-Rosales L. (2003). Bean common mosaic virus and Bean common mosaic necrosis virus in Mexico. Plant Dis..

[4] Feng X., Myers J.R., Karasev A.V. (2015). Bean common mosaic virus Isolate Exhibits a Novel Pathogenicity Profile in Common Bean, Overcoming the bc-3 Resistance Allele Coding for the Mutated eIF4E Translation Initiation Factor. Phytopathology.

[5] Feng X., Poplawsky A.R., Nikolaeva O.V., Myers J.R., Karasev A.V. (2014). Recombinants of bean common mosaic virus (BCMV) and genetic determinants of BCMV involved in overcoming resistance in common bean. Phytopathology.

[6] Martínez-Pérez M., Aparicio F., López-Gresa M.P., Bellés J.M., Sánchez-Navarro J.A., Pallás V. (2017). *Arabidopsis* m^6^A demethylase activity modulates viral infection of a plant virus and the m^6^A abundance in its genomic RNAs. Proc. Natl. Acad. Sci. USA.

[7] Zhang T., Shi C., Hu H., Zhang Z., Wang Z., Chen Z., Feng H., Liu P., Guo J., Lu Q. (2022). N6-methyladenosine RNA modification promotes viral genomic RNA stability and infection. Nat. Commun..

[8] Han C., Zhang F., Qiao X., Zhao Y., Qiao Q., Huang X., Zhang S. (2021). Multi-Omics Analysis Reveals the Dynamic Changes of RNA N^6^-Methyladenosine in Pear (*Pyrus bretschneideri*) Defense Responses to Erwinia amylovora Pathogen Infection. Front. Microbiol..

[9] Li B., Zhang M., Sun W., Yue D., Ma Y., Zhang B., Duan L., Wang M., Lindsey K., Nie X. (2023). N6-methyladenosine RNA modification regulates cotton drought response in a Ca^2+^ and ABA-dependent manner. Plant Biotechnol. J..

[10] Hu J., Cai J., Park S.J., Lee K., Li Y., Chen Y., Yun J.Y., Xu T., Kang H. (2021). N^6^-Methyladenosine mRNA methylation is important for salt stress tolerance in *Arabidopsis*. Plant J..

[11] Wang S., Wang H., Xu Z., Jiang S., Shi Y., Xie H., Wang S., Hua J., Wu Y. (2023). m6A mRNA modification promotes chilling tolerance and modulates gene translation efficiency in *Arabidopsis*. Plant Physiol..

[12] Tuck M.T. (1992). The formation of internal 6-methyladenine residues in eucaryotic messenger RNA. Int. J. Biochem..

[13] Zhou C., Molinie B., Daneshvar K., Pondick J.V., Wang J., Van Wittenberghe N., Xing Y., Giallourakis C.C., Mullen A.C. (2017). Genome-Wide Maps of m6A circRNAs Identify Widespread and Cell-Type-Specific Methylation Patterns that Are Distinct from mRNAs. Cell Rep..

[14] Rinn J.L., Chang H.Y. (2012). Genome regulation by long noncoding RNAs. Annu. Rev. Biochem..

[15] Bokar J.A., Shambaugh M.E., Polayes D., Matera A.G., Rottman F.M. (1997). Purification and cDNA cloning of the AdoMet-binding subunit of the human mRNA (N6-adenosine)-methyltransferase. RNA.

[16] Liu J., Yue Y., Han D., Wang X., Fu Y., Zhang L., Jia G., Yu M., Lu Z., Deng X. (2014). A METTL3-METTL14 complex mediates mammalian nuclear RNA N6-adenosine methylation. Nat. Chem. Biol..

[17] Ping X.L., Sun B.F., Wang L., Xiao W., Yang X., Wang W.J., Adhikari S., Shi Y., Lv Y., Chen Y.S. (2014). Mammalian WTAP is a regulatory subunit of the RNA N6-methyladenosine methyltransferase. Cell Res..

[18] Schwartz S., Mumbach M.R., Jovanovic M., Wang T., Maciag K., Bushkin G.G., Mertins P., Ter-Ovanesyan D., Habib N., Cacchiarelli D. (2014). Perturbation of m6A writers reveals two distinct classes of mRNA methylation at internal and 5′ sites. Cell Rep..

[19] Patil D.P., Chen C.K., Pickering B.F., Chow A., Jackson C., Guttman M., Jaffrey S.R. (2016). m^6^A RNA methylation promotes XIST-mediated transcriptional repression. Nature.

[20] Bawankar P., Lence T., Paolantoni C., Haussmann I.U., Kazlauskiene M., Jacob D., Heidelberger J.B., Richter F.M., Nallasivan M.P., Morin V. (2021). Hakai is required for stabilization of core components of the m^6^A mRNA methylation machinery. Nat. Commun..

[21] Wen J., Lv R., Ma H., Shen H., He C., Wang J., Jiao F., Liu H., Yang P., Tan L. (2018). Zc3h13 Regulates Nuclear RNA m^6^A Methylation and Mouse Embryonic Stem Cell Self-Renewal. Mol. Cell.

[22] Jia G., Fu Y., Zhao X., Dai Q., Zheng G., Yang Y., Yi C., Lindahl T., Pan T., Yang Y.G. (2011). N6-methyladenosine in nuclear RNA is a major substrate of the obesity-associated FTO. Nat. Chem. Biol..

[23] Zheng G., Dahl J.A., Niu Y., Fedorcsak P., Huang C.M., Li C.J., Vågbø C.B., Shi Y., Wang W.L., Song S.H. (2013). ALKBH5 is a mammalian RNA demethylase that impacts RNA metabolism and mouse fertility. Mol. Cell.

[24] Wang X., Lu Z., Gomez A., Hon G.C., Yue Y., Han D., Fu Y., Parisien M., Dai Q., Jia G. (2014). N6-methyladenosine-dependent regulation of messenger RNA stability. Nature.

[25] Xu C., Wang X., Liu K., Roundtree I.A., Tempel W., Li Y., Lu Z., He C., Min J. (2014). Structural basis for selective binding of m6A RNA by the YTHDC1 YTH domain. Nat. Chem. Biol..

[26] Wang X., Zhao B.S., Roundtree I.A., Lu Z., Han D., Ma H., Weng X., Chen K., Shi H., He C. (2015). N^6^-methyladenosine Modulates Messenger RNA Translation Efficiency. Cell.

[27] Zhong S., Li H., Bodi Z., Button J., Vespa L., Herzog M., Fray R.G. (2008). MTA is an *Arabidopsis* messenger RNA adenosine methylase and interacts with a homolog of a sex-specific splicing factor. Plant Cell.

[28] Shen L., Liang Z., Gu X., Chen Y., Teo Z.W., Hou X., Cai W.M., Dedon P.C., Liu L., Yu H. (2016). N^6^-Methyladenosine RNA Modification Regulates Shoot Stem Cell Fate in *Arabidopsis*. Dev. Cell.

[29] Růžička K., Zhang M., Campilho A., Bodi Z., Kashif M., Saleh M., Eeckhout D., El-Showk S., Li H., Zhong S. (2017). Identification of factors required for m^6^ A mRNA methylation in *Arabidopsis* reveals a role for the conserved E3 ubiquitin ligase HAKAI. New Phytol..

[30] Parker M.T., Knop K., Zacharaki V., Sherwood A.V., Tomé D., Yu X., Martin P.G., Beynon J., Michaels S.D., Barton G.J. (2021). Widespread premature transcription termination of *Arabidopsis thaliana* NLR genes by the spen protein FPA. elife.

[31] Zhang M., Bodi Z., Mackinnon K., Zhong S., Archer N., Mongan N.P., Simpson G.G., Fray R.G. (2022). Two zinc finger proteins with functions in m^6^A writing interact with HAKAI. Nat. Commun..

[32] Reichel M., Köster T., Staiger D. (2019). Marking RNA: m6A writers, readers, and functions in *Arabidopsis*. J. Mol. Cell Biol..

[33] Yue H., Nie X., Yan Z., Weining S. (2019). N6-methyladenosine regulatory machinery in plants: Composition, function and evolution. Plant Biotechnol. J..

[34] Li D., Zhang H., Hong Y., Huang L., Li X., Zhang Y., Ouyang Z., Song F. (2014). Genome-wide identification, biochemical characterization, and expression analyses of the YTH domain-containing RNA-binding protein family in *Arabidopsis* and Rice. Plant Mol. Biol. Rep..

[35] Hou Y., Sun J., Wu B., Gao Y., Nie H., Nie Z., Quan S., Wang Y., Cao X., Li S. (2021). CPSF30-L-mediated recognition of mRNA m^6^A modification controls alternative polyadenylation of nitrate signaling-related gene transcripts in *Arabidopsis*. Mol. Plant.

[36] Tian S., Wu N., Zhang L., Wang X. (2021). RNA N^6^-methyladenosine modification suppresses replication of rice black streaked dwarf virus and is associated with virus persistence in its insect vector. Mol. Plant Pathol..

[37] He H., Ge L., Li Z., Zhou X., Li F. (2023). Pepino mosaic virus antagonizes plant m^6^A modification by promoting the autophagic degradation of the m^6^A writer HAKAI. aBIOTECH.

[38] He H., Ge L., Chen Y., Zhao S., Li Z., Zhou X., Li F. (2024). m^6^A modification of plant virus enables host recognition by NMD factors in plants. Sci. China Life Sci..

[39] Zhang K., Zhuang X., Dong Z., Xu K., Chen X., Liu F., He Z. (2021). The dynamics of N^6^-methyladenine RNA modification in interactions between rice and plant viruses. Genome Biol..

[40] Su H., Meng L., Qu Z., Zhang W., Liu N., Cao P., Jin J. (2024). Genome-wide identification of the N^6^-methyladenosine regulatory genes reveals NtFIP37B increases drought resistance of tobacco (*Nicotiana tabacum* L.). BMC Plant Biol..

[41] Zheng H., Gao Y., Dang Y., Wu F., Wang X., Zhang F., Sui N. (2023). Characterization of the m6A gene family in sorghum and its function in growth, development and stress resistance. Ind. Crops Prod..

[42] Andrade M.A., Perez-Iratxeta C., Ponting C.P. (2001). Protein repeats: Structures, functions, and evolution. J. Struct. Biol..

[43] Wang J., Jiang Y., Sun T., Zhang C., Liu X., Li Y. (2022). Genome-Wide Classification and Evolutionary Analysis Reveal Diverged Patterns of Chalcone Isomerase in Plants. Biomolecules.

[44] Liu Y., Xiao L., Chi J., Li R., Han Y., Cui F., Peng Z., Wan S., Li G. (2022). Genome-wide identification and expression of SAUR gene family in peanut (*Arachis hypogaea* L.) and functional identification of AhSAUR3 in drought tolerance. BMC Plant Biol..

[45] Shen L. (2023). Functional interdependence of N6-methyladenosine methyltransferase complex subunits in *Arabidopsis*. Plant Cell.

[46] Zheng H.X., Sun X., Zhang X.S., Sui N. (2020). m^6^A Editing: New Tool to Improve Crop Quality?. Trends Plant Sci..

[47] Ren Z., Tang B., Xing J., Liu C., Cai X., Hendy A., Kamran M., Liu H., Zheng L., Huang J. (2022). MTA1-mediated RNA m^6^ A modification regulates autophagy and is required for infection of the rice blast fungus. New Phytol..

[48] Guo T., Liu C., Meng F., Hu L., Fu X., Yang Z., Wang N., Jiang Q., Zhang X., Ma F. (2022). The m^6^ A reader MhYTP2 regulates MdMLO19 mRNA stability and antioxidant genes translation efficiency conferring powdery mildew resistance in apple. Plant Biotechnol. J..

[49] Burow M.D., Chlan C.A., Sen P., Lisca A., Murai N. (1990). High-frequency generation of transgenic tobacco plants after modified leaf disk cocultivation with *Agrobacterium tumefaciens*. Plant Mol. Bio. Rep..

[50] Pflieger S., Blanchet S., Meziadi C., Richard M.M., Thareau V., Mary F., Mazoyer C., Geffroy V. (2014). The “one-step” Bean pod mottle virus (BPMV)-derived vector is a functional genomics tool for efficient overexpression of heterologous protein, virus-induced gene silencing and genetic mapping of BPMV R-gene in common bean (*Phaseolus vulgaris* L.). BMC Plant Biol..

[51] Zhang C., Bradshaw J.D., Whitham S.A., Hill J.H. (2010). The development of an efficient multipurpose bean pod mottle virus viral vector set for foreign gene expression and RNA silencing. Plant Physiol..

[52] Wang P., Doxtader K.A., Nam Y. (2016). Structural Basis for Cooperative Function of Mettl3 and Mettl14 Methyltransferases. Mol. Cell.

[53] Wang X., Feng J., Xue Y., Guan Z., Zhang D., Liu Z., Gong Z., Wang Q., Huang J., Tang C. (2016). Structural basis of N6-adenosine methylation by the METTL3–METTL14 complex. Nature.

[54] Hou N., Li C., He J., Liu Y., Yu S., Malnoy M., Mobeen Tahir M., Xu L., Ma F., Guan Q. (2022). MdMTA-mediated m^6^ A modification enhances drought tolerance by promoting mRNA stability and translation efficiency of genes involved in lignin deposition and oxidative stress. New Phytol..

[55] Zhou L., Tang R., Li X., Tian S., Li B., Qin G. (2021). N^6^-methyladenosine RNA modification regulates strawberry fruit ripening in an ABA-dependent manner. Genome Biol..

[56] Bodi Z., Zhong S., Mehra S., Song J., Graham N., Li H., May S., Fray R.G. (2012). Adenosine Methylation in *Arabidopsis* mRNA is Associated with the 3′ End and Reduced Levels Cause Developmental Defects. Front. Plant Sci..

[57] Zhang F., Zhang Y.C., Liao J.Y., Yu Y., Zhou Y.F., Feng Y.Z., Yang Y.W., Lei M.Q., Bai M., Wu H. (2019). The subunit of RNA N6-methyladenosine methyltransferase OsFIP regulates early degeneration of microspores in rice. PLoS Genet..

[58] Chen C., Chen H., Zhang Y., Thomas H.R., Frank M.H., He Y., Xia R. (2020). TBtools: An Integrative Toolkit Developed for Interactive Analyses of Big Biological Data. Mol. Plant.

[59] Lu S., Wang J., Chitsaz F., Derbyshire M.K., Geer R.C., Gonzales N.R., Gwadz M., Hurwitz D.I., Marchler G.H., Song J.S. (2020). CDD/SPARCLE: The conserved domain database in 2020. Nucleic Acids Res..

[60] Artimo P., Jonnalagedda M., Arnold K., Baratin D., Csardi G., de Castro E., Duvaud S., Flegel V., Fortier A., Gasteiger E. (2012). ExPASy: SIB bioinformatics resource portal. Nucleic Acids Res..

[61] Wang Y., Tang H., Debarry J.D., Tan X., Li J., Wang X., Lee T.H., Jin H., Marler B., Guo H. (2012). MCScanX: A toolkit for detection and evolutionary analysis of gene synteny and collinearity. Nucleic Acids Res..

[62] Koch M.A., Haubold B., Mitchell-Olds T. (2000). Comparative evolutionary analysis of chalcone synthase and alcohol dehydrogenase loci in *Arabidopsis*, *Arabis*, and related genera (Brassicaceae). Mol. Biol. Evol..

[63] Kumar S., Stecher G., Li M., Knyaz C., Tamura K. (2018). MEGA X: Molecular Evolutionary Genetics Analysis across Computing Platforms. Mol. Biol. Evol..

[64] Robert X., Gouet P. (2014). Deciphering key features in protein structures with the new ENDscript server. Nucleic Acids Res..

[65] Aik W., Scotti J.S., Choi H., Gong L., Demetriades M., Schofield C.J., McDonough M.A. (2014). Structure of human RNA N⁶-methyladenine demethylase ALKBH5 provides insights into its mechanisms of nucleic acid recognition and demethylation. Nucleic Acids Res..

[66] Xu C., Liu K., Ahmed H., Loppnau P., Schapira M., Min J. (2015). Structural Basis for the Discriminative Recognition of N6-Methyladenosine RNA by the Human YT521-B Homology Domain Family of Proteins. J. Biol. Chem..

[67] Śledź P., Jinek M. (2016). Structural insights into the molecular mechanism of the m^6^A writer complex. elife.

[68] Bailey T.L., Johnson J., Grant C.E., Noble W.S. (2015). The MEME Suite. Nucleic Acids Res..

[69] Rombauts S., Déhais P., Van Montagu M., Rouzé P. (1999). PlantCARE, a plant cis-acting regulatory element database. Nucleic Acids Res..

[70] Szklarczyk D., Gable A.L., Nastou K.C., Lyon D., Kirsch R., Pyysalo S., Doncheva N.T., Legeay M., Fang T., Bork P. (2021). The STRING database in 2021: Customizable protein-protein networks, and functional characterization of user-uploaded gene/measurement sets. Nucleic Acids Res..

[71] Livak K.J., Schmittgen T.D. (2001). Analysis of relative gene expression data using real-time quantitative PCR and the 2(-Delta Delta C(T)) Method. Methods.

[72] Dominissini D., Moshitch-Moshkovitz S., Salmon-Divon M., Amariglio N., Rechavi G. (2013). Transcriptome-wide mapping of N^6^-methyladenosine by m^6^A-seq based on immunocapturing and massively parallel sequencing. Nat. Protoc..

[73] Ratel D., Ravanat J.L., Berger F., Wion D. (2006). N6-methyladenine: The other methylated base of DNA. Bioessays.

[74] Schmittgen T.D., Livak K.J. (2008). Analyzing real-time PCR data by the comparative C(T) method. Nat. Protoc..

[75] Pflieger S., Blanchet S., Camborde L., Drugeon G., Rousseau A., Noizet M., Planchais S., Jupin I. (2008). Efficient virus-induced gene silencing in *Arabidopsis* using a ‘one-step’ TYMV-derived vector. Plant J..

