# Supplementary material for: Characterization of the m6A Regulatory Gene Family in Phaseolus vulgaris L. and Functional Analysis of PvMTA in Response to BCMV Infection
Source: Int J Mol Sci. 2025 Mar 19;26(6):2748. doi: 10.3390/ijms26062748 (PMC11942742; doi:10.3390/ijms26062748)
Supplement: Supplementary file 1 [file ijms-26-02748-s001.zip › Supplementary Tables.pdf]

Supplementary Table S1. Gene ID and transcript ID of identified m6A regulatory genes in common bean.

Gene ID	Transcript ID
Phvul.010G102500	Phvul.010G102500.1
Phvul.007G073300	Phvul.007G073300.1
Phvul.001G016200	Phvul.001G016200.1
	Phvul.001G016200.2
Phvul.001G262100	Phvul.001G262100.1
Phvul.009G262600	Phvul.009G262600.1
Phvul.001G131400	Phvul.001G131400.1
Phvul.006G137400	Phvul.006G137400.1
Phvul.006G137611	Phvul.006G137611.1
Phvul.004G131600	Phvul.004G131600.1
Phvul.008G264300	Phvul.008G264300.1
Phvul.002G123600	Phvul.002G123600.1
	Phvul.002G123600.2
Phvul.001G044000	Phvul.001G044000.2
Phvul.006G214800	Phvul.006G214800.1
	Phvul.006G214800.2
Phvul.001G147800	Phvul.001G147800.1
Phvul.007G168900	Phvul.007G168900.1
Phvul.002G181800	Phvul.002G181800.1
	Phvul.002G181800.2
Phvul.003G014200	Phvul.003G014200.1
Phvul.001G110200	Phvul.001G110200.1
Phvul.002G247000	Phvul.002G247000.1
Phvul.004G080300	Phvul.004G080300.1
	Phvul.004G080300.2
Phvul.010G165400	Phvul.010G165400.1
	Phvul.010G165400.2
	Phvul.010G165400.3
	Phvul.010G165400.4
	Phvul.010G165400.5
	Phvul.010G165400.6
Phvul.003G119300	Phvul.003G119300.1
	Phvul.003G119300.2
	Phvul.003G119300.3
	Phvul.003G119300.4
	Phvul.003G119300.5
	Phvul.003G119300.6
Phvul.006G121600	Phvul.006G121600.1
Phvul.005G045600	Phvul.005G045600.1
Phvul.004G132700	Phvul.004G132700.1
Phvul.006G218800	Phvul.006G218800.1
Phvul.002G152600	Phvul.002G152600.1
	Phvul.002G152600.2
	Phvul.002G152600.3

	Phvul.002G152600.4
	Phvul.002G152600.5
	Phvul.002G152600.6
	Phvul.002G152600.7
	Phvul.002G152600.8
	Phvul.002G152600.9
	Phvul.002G152600.10
	Phvul.002G152600.11
	Phvul.002G152600.12
	Phvul.002G152600.13
	Phvul.002G152600.14
	Phvul.002G152600.15
	Phvul.002G152600.16
	Phvul.002G152600.17
	Phvul.002G152600.18
	Phvul.002G152600.19
	Phvul.002G152600.20
Phvul.006G130200	Phvul.006G130200.1
Phvul.002G107400	Phvul.002G107400.1
Phvul.007G267500	Phvul.007G267500.1
Phvul.008G108800	Phvul.008G108800.1

Supplementary Table S2. Gene name, accession numbers and amino acid sequences used in this study.

Species name	Genes name	Accession numbers	Amino acid sequences
Arabidopsis thaliana	AtMTA	AT4G10760	METESDDATITVVKDMRVRLENRIRTQHDAHLDLLSSLQSIVPDIVPSLDLSLKLISSFTNRPFVATPPLPEPKVEKKHHPIVKLGTQLQ QLHGHDSKSMLVDSNQRDAEADGSSGSPMALVRAMVAECLLQRVPFSPTDSSTVLRKLENDQNARPAEKAALRDLGGECGPILAV ETALKSMAEENGSVELEEFEVSGKPRIMVLAIDRTRLLKELPESFQGNNESNRVVETPNSIENATVSGGGFGVSGSGNFPRPEMWGG DPNMGFRPMMNAPRGMQMMGMHHPMGIMGRPPPFPLPLPLPVPSNQKLRSEEEDLKDVEALLSKKSFKEKQQSRTGEELLDLIHRP TAKEAATAAKFKSKGGSQVKYYCRYLTKEDCRLQSGSHIACNKRHFRRLIASHTDVSLGDCSFLDTCRHMKTCKYVHYELDMADA MMAGPDKALKPLRADYCSEAELGEAQWINCDIRSFRMDILGTFGVVMADPPWDIHMELPYGTMADDEMRTLNVPSLQTDGLIFLW VTGRAMELGRECLELWGYKRVEEIIWVKTNQLQRIIRTGRTGHWLNHSKEHCLVGIKGNPEVNRNIDTDVIVAEVRETSRKPDEMY AMLERIMPRARKLELFARMHNAHAGWLSLGNQLNGVRLINEGLRARFKASYPEIDVQPPSPPRASAMETDNEPMAIDSITA
	AtMTB	AT4G09980	MKKKQEESSLEKLSTWYQDGEQDGGDRSEKRRMSLKASDFESSSRSGGSKSKEDNKSVVDVEHQDRDSKRERDGRERTHGSSSDS SKRKRWDEAGGLVNDGDHKSSKLSDSRHDSGGERVSVSNEHGESRRDLKSDRSLKTSSRDEKSKSRGVKDDDRGSPLKKTSGKDG SEVVREVGRSNRSKTPDADYEKEKYSRKDERSRGRDDGWSDRDRDQEGLKDNWKRRHSSSGDKDQKDGDLLYDRGREREFPRQG RERSEGERSHGRLGGRKDGNRGEAVKALSSGGVSNENYDVIEIQTKPHDYVRGESGPNFARMTESGQQPPKKPSNNEEEWAHNQE GRQRSETFGFGSYGEDSRDEAGEASSDYSGAKARNQRGSTPGRTNFVQTPNRGYQTPQGTRGNRPLRGGKGRPAGGRENQQGAIP MPIMGSPFANLGMPPPSPIHSLTPGMSPIPGTSVTPVFMPPFAPTLIWPGARGVDGNMLPVPPVLSPLPPGPSGPRFPSIGTPPNPNMFF TPPGSDRGGPPNFPGSNISGQMGRGMPSDKTSGGWVPPRGGGPPGKAPSRGEQNDYSQNFVDTGMRPQNFIRELELTNVEDYPKLR ELIQKKDEIVSNSASAPMYLKGDLHEVELSPELFGTKFDVILVDPPWEEYVHRAPGVSDSMEYWTFEDIINLKIEAIADTPSFLFLWVG DGVGLEQGRQCLKKWGFRRCEDICWVKTNKSNAAPTLRHDSRTVFQRSKEHCLMGIKGTVRRSTDGHIIHANIDTDVIIAEEPPYGS TQKPEDMYRIIEHFALGRRRLELFGEDHNIRAGWLTVGKGLSSSNFEPQAYVRNFADKEGKVWLGGGGRNPPPDAPHLVVTTPDIES LRPKSPMKNQQQQSYPSSLASANSSNRRTTGNSPQANPNVVVLHQEASGSNFSVPTTPHWVPPTAPAAAGPPPMDSFRVPEGGNNT RPPDDKSFDMYGFN
	AtMTC	AT1G19340	MAKTDKLAQFLDSGIYESDEFNWFFLDTVRITNRSYTRFKVSPSAYYSRFFNSKQLNQHSSESNPKKRKRKQKNSSFHLPSVGEQAS NLRHQEARLFLSKAHESFLKEIELLSLTKGLSDDNDDDDSSLLNKCCDDEVSFIELGGVWQAPFYEITLSFNLHCDNEGESCNEQRVF QVFNNLVVNEIGEEVEAEFSNRRYIMPRNSCFYMSDLHHIRNLVPAKSEEGYNLIVIDPPWENASAHQKSKYPTLPNQYFLSLPIKQL AHAEGALVALWVTNREKLLSFVEKELFPAWGIKYVATMYWLKVKPDGTLICDLDLVHHKPYEYLLLGYHFTELAGSEKRSDFKLL DKNQIIMSIPGDFSRKPPIGDILLKHTPGSQPARCLELFAREMAAGWTSWGNEPLHFQDSRYFLKV
	AtALKBH1A	AT1G11780	MYESANVSDDADRTAFRRAEKKYKLYYEQDSKFSRKKKLPKPIDLSELLDFNLISQNFNNDGVLPDGIRVSKVDSSPVFCIDNRPGF YFIPDALSLKEQCKWIKESLTSFPQPPNRTNHNAIYGPIDDLFDSAKENKVLVQDDLTNNKWKFYEEVDIEKATRSSCKSVSASVLLR KLRWSTLGLQFDWSKRNYDVSLPHNNIPDALCQLAKTHAAIAMPDGEEFRPEGAIVNYFGIGDTLGGHLDDMEADWSKPIVSMSLG CKAIFLLGGKSKDDPPHAMYLRSGDVVLMAGEARECFHGIPRIFTGEENADIGALESELSHESGHFFAEYIKTSRININIRQVF
	AtALKBH1B	AT3G14140	MATTNYSMIFIGNLQVAVDRSSLYESSDYSSFVARECLNKDSAMKPYDIFIFEKDEHFSVEDHYTAGNSSNESNTRPSDIFLKKKLVG LNPSILQLNREKRKATLKFSGDNKDILQSTLSSSKNINLSGGLFVSREYENKYVKFGARMRCDLVNRMKDVTLAWHESVSSTSDKN VELSSVENHKIAPKADGPGNSSNESSSSPFDIFLKKKVMRLKPSFLELNREKKKAAKGFSGIVIRPGMVLLKNYLSINNQVMIVNKCR QLGLGEGGFYQPGFQDGGLLHLKMMCLGKNWDCQTRRYGEIRPIDGSVPPRIPVEFSQLVEKAIKESKSLVATNSNETKGGDEIPLL LPDICVVNFYTSTGKLGLHQVSVYDKTSFDFLKYKGGYLNTDKGESKKSLRKGLPIVSFSIGDSAEFLYGDQKDVDKADTLILESGD VLIFGERSRNVFHGVRSIRKILPPRLFFRKQIFNQVV
	AtALKBH1C	AT3G14160	MNHSEARGSGGHKGYRGRSQASEQWVPVAADDKSLEDSSGNRVRGSQGKGRSRTSWSPRNSYGRGNDEHSPVQAYVNKSNVGF VEKGVQQDRKSLEDGIGSTKQPDDGAAAGDNKAVLQSKSTNVSGGSFVSSECEDKDGAKMYCDLVNRVNDVTLSCQESVSSTVV QKVELSSVEDQKSAPKADGAGNSSNESSTRHFDIFLEKKGIVLKPNLLVLSREKKKAAKGYSGTVIRPGMVLLKNYLSINDQVMIVN KCRRLGLGEGGFYQPGYRDEAKLHLKMMCLGKNWDPETSRYGETRPFDGSTAPRIPAEFNQFVEKAVKESQSLAASNSKQTKGGD EIPFMLPDICIVNFYSSTGRLGLHQDKDESENSIRKGLPVVSFSIGDSAEFLYGDQRDEDKAETLTLESGDVLLFGGRSRKVFHGVRSI RKDTAPKALLQETSLRPGRLNLTFRQY
	AtALKBH1D	AT5G01780	MLNSIHRSFHLTSRHQIVLRLCSPSASRTIMSSSPHSPIPKTHTASLDSLNDYEEQFPPLTGGSKTKKFYLGSTNPSTPCQSSQLQNWTS GKDALSLQRNLGCKNRRRRRASRFLHEESNGTTFEVGAGIGSPTSMVHFDSTNPSSSSKSSQSQNLKIRKVRNHRNSGFKSRDQSPQ RIKDPPPFDICSSVLERNDTSIKDWILADETNRETVEVSNKHKVIRPGMVLLKDFLTPDIQVDIVKTCRELGVKPTGFYQPGYSVGSKL

			HLQMMCLGRNWDPQTKYRKNTDIDSKAPEIPVTFNVLVEKAIREAHALIDRESGTEDAERILPVMSPDICIVNFYSETGRLGLHQDR<br>DESEESIARGLPIVSFSIGDSAEFLYGEKRDVEEAQGVILESGDVLIFGGESRMIFHGVKSIIPNSAPMSLLNESKLRTGRLNLTFRHF
	AtALKBH2	AT2G22260	MWFSSQNGFLVPSLFNVSYRKNCSGHKLKEVTDSDTFSDNGKDDSDTKKRFHYHQDQRRMSLTSIVAVESPSSSNAPSRKTIDLGH<br>GSDLIYIQRFLPFQQSWTFFDYLDKHIPWTRPTIRVFGRSCLQPRDTCYVASSGLTALVYSGYRPTSYSWDDFPPLKEILDAIYKVLPG<br>SRFNSLLLNRYKGASDYVAWHADDEKIYGPTPEIASVSFGCERDFVLKKKKDEESSQGKTGDSGPAKKRLKRSSREDQQSLTLKHG<br>SLLVMRGYTQRDWIHSVPKRAKAEGTRINLTFRLVL
	AtALKBH6	AT4G20350	MKRVLFHKSVMPPWLTKITAEIHESSGLFPSAINHVLINEYHPDQGIMPHQDGPAYFPVVAILSLGSPVVMDFTPHLRLRSGDGYISK<br>DQSPCAESCAPERDSFSVLLMPQSLLIFKDDAYSDFLHGISDSPTQCYNQVVNEAEALAYSNEEDSRKDGDKIFHRDQTRVSLTCRLV<br>PKVRKNLFRF
	AtALKBH7	AT4G02485	MDEEAENLRAAFGDSSDDEDIADRPGKETIGIGDSAVWERVEEINGLWLYRNFLSIAHQSHLLSAILNEGWFVEESINQAMRFGDLPS<br>WATELSDLIRETLESVDLPVLSADLLWREPLFDQLIVNLYQPGEGICAHVDLLRFEDGIAIVSLESPCVMRFSPAEKNEYEAVDVLLN<br>PGSLILMSGEARYRWKHEINRKQNGFQLWEGEEIDQKRRISITLRKLCQA
	AtALKBH8	AT1G31600	MGWPWADHWTMVLNGLGQIFRPLSIKDQPLEPMKKMFQVDMTIGLWTAQLTLLTQVNVIVFFIEHIGEDTSPLYDRRKLQSFIPRM<br>VQPRFVRPTQSSPSSISGEPNSSNLYVANCGPAVGLTHNAIAAVFAEFGEVNGVYAADDSGVRVIVSFADPFSAKAALEALSGRPCPD<br>LKGRSLHIRYSVLQLPSETQVNDCVPVSLIDSELNIPGLFLLPDFVTVAEEQQLLAAVDARHWIGLAKRRVQHYGYEFCYGTRNVDT<br>KKRLGELPSFVSPILERIYLFPNFDNGSASLNLDQLTVNEYPSGVGLSPHIDTHSAFEDCIFSLSLAGPCIMEFRRYSVSTWKASTTDAE<br>KSGDSSCIKKALYLPPRSMLLLSGEARYAWNHYIPHHKIDKVKDKVIRRSSRRVSFTLRKVRNHPCSCKYPQYCDSQQQM
	AtALKBH9A	AT1G48980	MDNYSFIRQSELEIASKVNLLSKRIRSQEQEVAVDFSHMTLEDELSEEEDHKDSSREAFGSSLENHKLSRKQRTHIRAINVKRKRDFV<br>CLEKVNGELVNILEGLELHTEVFNAAEQRRIVDKVCELQEKVQKGELKRAFTAQGKGRSTIQFGCCFNYRTSKTGNLAGILKHETVD<br>PLPHLFKVIIRRLVKWHVLPPTCVPDCCVVNIYDEGDCIPPHIDHHDFLRPFCTVSFLSECNILFGSNLKVEETGEYSGGSYSLPLPVGS<br>VLVLNGNGADVAKHCVPEVPTKRKMDESKWPVWYTPEPYLQGIQPLTYELKSCGSSDHVF
	AtALKBH9B	AT2G17970	MENDPFLRQYQPSELKIASEFLTNWLPFLSKDLCKDCNHLLSNRIRSLDPAHCSNNTDKVDGECKTGSCSVVENMGSERASNNVDD<br>NYDEKSENGEDCDNHSLGSWKGSEIVFGSFPEDFSSVLQSRPAVVETASPRMRWADMAQEDEFDEEEEEEEEERDSSRKGFDASSM<br>KTPEKPKLSRDQRENLRLINVKRKKDFICLERVKGKIVNVLDGLELHTGVFSAVEQKRIVDQVYQLQEKGRRGELKKRTFTAPHKW<br>MRGKGRETIQFGCCYNYAPDRAGNPPGILQREEVDPLPHLFKVIIRKLIKWHVLPPTCVPDSCIVNIYDEGDCIPPHIDNHDFLRPFCTI<br>SFLSECDILFGSNLKVEGPGDFSGSYSIPLPVGSVLVLNGNGADVAKHCVPAVPTKRISITFRKMDESKRPVWFTPEPDLQGIEPLPLD<br>LNRSGSTSRFSRLNNHNGTNQRGHGRRGGGNGYDSRGYYNPERSSEHNDSGDWPSSQRRGMPRPSRRNYG
	AtALKBH9C	AT4G36090	MEPNYEEDVFLAKYQSSELKIASEFLTNWLPFLSRDLCNDCAHVLSDRIRSLDPEHCSNGEVKAGSGSMVDNMESEEVDNMESEEV<br>DNKMDNIYDGKLCVSHSLDSLKAGAFVFGSLSDSVSHAMPSSQSTVSEAASAQMSWADMGEEDGLEEEDQKENELGSHGVDVSPS<br>VGDSMKTPEKRKLSREERERYRFMNVKKMKVFSCYEKVRGRSVNILEGLELHTGVFSAVEQKKIVDFVYELQEKGRRGELRERTFT<br>APHKWMRGKGRVTIQFGCCYNYAPDKAGNPPGILQRGDVDPMPSIFKVIIKRLVGWHVLPPTCVPDSCIVNIYEEDDCIPPHIDNHDF<br>LRPFCTVSFLSECNILFGSNLKVLGPGEFSGSYSIPLPVG
	AtALKBH10A	AT2G48080	MAETPASPPLHPPVVLLSDSAAKDAMLTWFRGEFAAANAIIDALCAHLMQASGGSAQYESVMAALHRRRLNWIPVLQMQKYHSIS<br>QVTLQLQQHLAKGFHHHLDDDHDDDSPSSDITDGGSREEETLSICCKHEDECESRGASLLKQSKRFSAKEHVRGHTANVVKGLKLY<br>QDVFTRPQLSKLLDSINQLREAGRNHQLSGETFVLFNKNTKGTKRELLQLGVPIFGNTTDEHSVEPIPTLVQSVIDHLLQWRLIPEYKR<br>PNGCVINFFDEDEHSQPFQKPPHVDQPISTLVLSESTMVFGHRLGVDNDGNFRGSLTLPLKEGSLLVMRGNSADMARHVMCPSPNK<br>RVAITFFKLKPDSGKVQPPPTLWRPGTPSPLVMLAPAPKRLDAGTGVFLPWTPPVSRKPAKHLPPRVQRLRLLSSSKSVADSESSSPEI<br>GVS
	AtALKBH10B	AT4G02940	MTIAAAPARQTDRSATGFNPAYVTTAKAVSVPVQVPPATVVSEGLGKDALISWFRGEFAAANAIIDAMCSHLRIAEEAVSGSEYEAV<br>FAAIHRRRLNWIPVLQMQKYHSIAEVAIELQKVAAKKAEDLKQKKTEEEAEEDLKEVVATEEEEVKKECFNGEKVTENDVNGDVE<br>DVEDDSPTSDITDSGSHQDVHQTVVADTAHQIICHSHEDCDARSCEIKPIKGFQAKEQVKGHTVNVVKGLKLYEELLKEDEISKLLDF<br>VAELREAGINGKLAGESFILFNKQIKGNKRELIQLGVPIFGHVKADENSNDTNNSVNIEPIPPLLESVIDHFVTWRLIPEYKRPNGCVIN<br>FFEEGEYSQPFLKPPHLEQPISTLVLSESTMAYGRILSSDNEGNFRGPLTLSLKQGSLLVMRGNSADMARHVMCPSQNKRVSITFFRIR<br>PDTYHNHSQPNSPRNDGVMTMWQPYQMTPTPFLNGYDHSIDMMPKLGVLRPPMVMMAPPPVQPMILPSPNVMGTGGGTGVFLPW<br>ASVNSSRKHVKHLPPRAQKKRLLPLPPAASSSPAGGSTSEPVITVG
	AtALKBH10C	AT1G14710	MAMPPPGNVTTPSEKLQFPPPANWIPDERDGFISWLRAEFAAANAIIDSLCQHLQAVGDHNEYESVIGSIHHRRLAWSQVLTMQQFF<br>PVADVSYNLQQIAWKRQQQMPPQRHYNSDQVGKFGARRSGPGFNKHHGGGGGYRGADSMARNGHNFNGVNSDRVEHREEAKL<br>ASDVKALSVAEEKRDGSEKPRSDSKVEKKLEESETQEEIVKNHKCNSGSKDNSLISEQKQEENDKECPASMAKTFVVQEMYDAKM<br>VNVVEGLKLYDKMLDANEVSQLVSLVTNLRLAGRRGQLQSEAYVGYKRPNRGHGREMIQLGLPIADTPPDDDSIKDRRIEPIPSALS

			DIIERLVSKQIIPVKPDACIIDFFSEGDHSQPHMFVPWFGRPISVLSLSECDYTFGRVIVSENPGDYKGSLKLSLTPGSVLLVEGKSANLAKYAIHATRKQRILISFIKSKPRNSNWGPPPSRSPNQHIRHPTGPPKHYPVVIPSTGVLPTPSHRPPNGAVQPIFIPPSPPLASPMPFPGGVPTGPPVWPLLPPHPRHQTAPQRPMPIPGTGVFLPPGSNQELADNSNGTEGKLDLKAKEEARNGFGEGECDGSNGKQSN
	AtECT1	AT3G03950	MAGAASSDRLVTSFPLLDTADLFQDLSLGSDANEVPMNFTKGSFQHPYGHAPYGASSHGSERRPNMNAGNLLNGGDSIGSYPWGYIPANYPSGGYPDPRFGYDRNSNHSSFSHLMNPHSSQEVPSFDQLGYNDHLYSNHGLYGLYGNVIDSGHAYGTFGYDSWKLGRGWYPVDGYRKTRSFNHGRGYSDEKADRLNELCRGPRSSDFKNPQVLNSSMLDAMKQDVSAVDLQRYNGENFPESFVKAKFFVIKSYSEDDVHNCIKYGAWSSTPTGNKKLNAAYYEAKENSQECPVYLLFSVNASGQFVGLAEMVGPVDFNKTMEYWQQDKWIGCFPVKWHIIKDIPNSLLRHITLANNENKPVTNSRDTQEVNLEHGTKIIKIFKEYMSKTCILDDYKFYETRQKIIRDKKIKQKKQALDGASGETINLS
	AtECT2	AT3G13460	MATVAPPADHLLQKLSLDSPAKASEIPEPNKKTAVYQYGGVDVHGQVPSYDRSLTPMLPSDAADPSVCYVPNPYNPYQYYNVYGSGQEWTDYPAYTNPEGVDMNSGIYGENGTVVYPQGYGYAAYPYSPATSPAPQLGGEGQLYGAQQYQYPNYFPNSGPYASSVATPTQPDLSANKPAGVKTLPADSNNVASAAGITKGSNGSAPVKPTNQATLNTSSNLYGMGAPGGGLAAGYQDPRYAYEGYYAPVPWHDGSKYSDVQRPVSGSGVASSYSKSSTVPSSRNQNYRSNSHYTSVHQPSSVTGYGTAQGYYNRMYQNKLYGQYGSTGRSALGYGSSGYDSRTNGRGWAATDNKYRSWGRGNSYYYGNENNVDGLNELNRGPRAKGTKNQKGNLDDSLEVKEQTGESNVTEVGEADNTCVVPDREQYNKEDFPVDYANAMFFIIKSYSEDDVHKSIKYNVWASTPNGNKKLAAAYQEAQQKAGGCPIFLFFSVNASGQFVGLAEMTGPVDFNTNVEYWQQDKWTGSFPLKWHIVKDVPNSLLKHITLENNENKPVTNSRDTQEVKLEQGLKIVKIFKEHSSKTCILDDFSFYEVRQKTILEKKAKQTQKQVSEEKVTDEKKESATAESASKESPAAVQTSSDVKVAENGSVAKPVTGDVVANGC
	AtECT3	AT5G61020	MANPDHVSDVLHNLSIDPTTKALAPDSETKLQGAYGGNGNDFLLNDELVEATKIGKPSLLSKDGGVTKDKGSNLKKLGYQSAAYNAKGSYGKGAYAYGYYPPAYQYPRHGYTGSYASGKTNLQYQYLTQQGRSAGNGQSYGGYMDNIYSNYGMCGPYTNGYGYGSYGYDSWKYMPNWYAVNNTYKPRNGYHGYGKENIEGLNEMNRGPRAKGFNSQDGSKVMAVSLKEQRVTETEKLSEDVSLLDPKDYNKIDFPETYTEAKFYVIKSYSEDDIHKSIKYSVWSSTPNGNKKLDASYNEAKQKSDGCPVFLLFSVNTSGQFVGLAEMVGPVDFNKTVEYWQQDKWIGCFPVKWHFVKDIPNSSLRHITLENNENKPVTNSRDTQEVKLEQGIKVIKIFKDHASKTCILDDFEFYENRQKIIQERKSKHLQIKKQTLVANADKGVMSKINLVKPQESTTASEDAAALGVAAEVTKESKVVKETELPVEKNAVATAC
	AtECT4	AT1G55500	MSTVAPPADQAADVLKKLSLDSKSRTLEIPEPTKKTGVYQYGAMDSNGQVPSFDRSLSPMLPSDALDPSVFYVPNVYQQPYYYGYGSDYTGYTNSESVDMTSGAYGENASLVYPQGYGYAAFPYSPATSPAPQLGGDGQLYGAQQYQYPFPLTASSGPFASSVPASTQSKLSTNKAANSASAGIPKGMNGSAPVKPLNQSALYGNSALGGGLAAGYQDPRYSYDGFYTPVSWHDGSNFSDVQRSVSGSGVASSYSKANNNVPATRNQNSSSNSHYTSMYQPASMTGYAAQGYYDRVSPNKSYGQYGSTVRSGMGYGSSGYGSRTNERGWLNTDNKYRSRGRGNSYFYGNENIDGLNELNRGPRAKGTKATEEVSSEEVKKQTFDESNTEETVTCVLPDREECNRDDFPVEYKDAKFFIIKSYSEDDVHKSIKYNVWASTPNGNKKLDAAYQEAQQKSSGCPVFLFFSVNASGQFIGLAEMKGPVDFNKNIEYWQQDKWTGSFPLKWHILKDVPNSLLKHITLEYNENKPVTNSRDTQEVKLEQGLKVVKIFKEHNSKTCILDDFSFYEARQKTILEKKAKQQQSQKQVWEGKTNDEKPGTVDSTM
	AtECT5	AT3G13060	MKEQTVPANNETSASFKSSQEAPVVVHPAKVAPLTGPYGLAGDFAGHLPSSILSPQAQGFYYRGYENPTGEWDEYSSYVNVEGLDITSPVGFNENASLVYQTGYGYNPQMPYGPYSPAASPLPSEGQLYSPQQFPFSGASPYYQQVVPPSMQYITSPTQPELTSLVGVDQQGDNIGPRQSYHPHPIGPFNGNQPNLGFPEWQQGFDGGIWSDWSKPSDMHRHSSSISPALSPQPLGSYGSYGGQNIPMGSQRQRSFYGFGSGSNSYNRGYMHSGGRGQGSNYGSRLISNVGMGNQGWIGVDNSRGRGRVSDPSLGGAYNGTFDILNEQNRGPRASKPKTQVLEELDSAADSKKNNKGSAKEHEESNNADFVTDYTNAKLFIIKSYSEDNVHKSIKYNVWASTPNGNKKLDAAYREAKDEKEPCPLFLLFSVNASSQFCGVAEMVGPVDFEKSVDYWQQDKWSGQFPVKWHIIKDVPNSQFRHIILENNDNKPVTNSRDTQEVKLEQGIEMLKIFKNYDADTSILDDFGFYEEREKIIQDRKARRQPSLPSAGVVAGENEHKPASAALPTDFMKNMSKSFAQVVRLDEGSKEAVKASSSPDAITTAAVSSGQSN
	AtECT6	AT3G17330	MYTEGASDFVIDQSMYYPAYYSSGYDSSVGLQGGQNENAPYICYTPSYGYAQSPYNPYNPYIPGASIGVDSSFVGFQQYYSDPPYESAASSPTYVPYVIQPDMVSNSSTDSLVANGGQSDGRGSMQRNGSAIAGLPKDAPKSTTTGQYKQPGIPKNVSTTASAHSLQGKTAYANTLLPYGKSDIANGVSSIASYSYKPCSKIYDARGDNNTTGSTYTSEQNRGSRTRRSRNQLIVKAYTTKAGNADAEGNIVINPDRYNKEDFSIEYSDARFFVIKSYSEDDVHKSIKYGVWSSTLNGNKKLQSVYEDAQRIATEKSRECPIFLFFSVNSSGLFCGVAEMTGPVSFDRDMDFWQQDKWSGSFPVKWHIIKDVPNSYFRHIILHNNENKPVTNSRDTQEIILKQGLEVLKLFKHHAEKTSLLDDFMYYEDRQRLMQEERARLPFRTFRRPFPVLNLDFSDRSKKSSKDVVKKPSVTSAETKVVQLKNSDGDEKSNTQEATDDSTPSTLKFGSLAIKPTAGTTFNPTQPKPKPTPSLGSDHKSDSSEEVTGSLADDIVSVGSLPIKVKGSKELSSKIAAVGTSPLSTQDQFRSKPGF
	AtECT7	AT1G48110	MYTSEAAPDFVVDQGMYYPVDASYGYYCTGYESPGDWENHQMFFGVDGSEVQYTGGQNENSPYICYTPSYGYAQSPYNPFNPYIPGASIGVDSAFVAPQQFYSIPPYQSVATSPTFVPYAIQPEIVSNSSTNSLVETGSANRGRSDGRGSRQRSGTATAGLQRNDPKLPAGNSLGKISEKPRPNSGQSRQSEMDKSDSTSSSGQARQGRVTSVSAQPVDVVSSSRVSSFRQLDIAPPQLNDFSKIATNNNNIRPKLYGGHANIIPDTVREQNRGRRSRALGNQLIVKAYTTKAGNADAEGNIVINPSQYNKEDLRIDYSNAKFFVIKSYSEDDVHKSIKYNVWSSTLHGNKKLQSAYEDAQRIATEKSCECPIFLFFSVNASGLFCGMAEMTGPVSFDKDMDFWQQDKWSGSFPVKWHIIKDVPNSYFRHIILQNNE

			NKPVTNSRDTQEIMLKQGLEVLKIFKDHMERTSLLDDFVYYESRQRVMQDERTRLPYRTFLNDLPLPRPDLSDRNKKTPLESFKKPS<br>VISAKTEELPSKSEGNEETTVKEGNEEDTSSTQKKISSLTIDPSGTDSNPTTVSHLNQKSQAKSKPNSSGSLKKTDPSEVVDASLSDKN<br>DSFKVTGSPAILTVGTIPLDPKSLQK
	AtECT8	AT1G79270	MVKNLKVDPLAKVTASTTSMVSAKENKNQSEAEPSYYETLETYQGLPCPYGGYYGYYYPGLDGSVGEAKDNGYYGYGTEVQYPV<br>MQGENGSVIYLMPGFQSYDASQTYMPINPVGVSSQALHSPMYAAQGYYQNQFGYADVSSPTYLWDPVGDRYVYGVASYTPPLKQ<br>NISSSSHNHNNYYSKSKNSFTGHGMGDRPKTPRKASQNSYAPPPLLNQEKGRIAYPMDPVKKKSGALNRDETEKAKARTKENGTSM<br>NDLANGQDHITNGECESCSLDAEGNERSNGVGSVIRRDQYNLPSFQTKYEEAIFFVIKSYSEDDIHKSIKYNVWSSTLNGNKKLDSAY<br>QESQKKAADKSGKCPVFLFFSVNASGQFCGVAEMIGRVDYEKSMEFWQQDKWTGYFPVKWHIIKDVPNPQLRHIILENNENKPVTN<br>SRDTQEVRLPQGNEVLNIFKNYAAKTSILDDFDFYENREKVMVQKKLRFPPVLKKKEEDLVADFKTMEMSNTVEEGNTELTGTVS
	AtECT9	AT1G27960	MAASYRSTDWFPPTTTTGSFTGLNTDQSVLQDQDIVSSRPFCGQGTESFHIGADTPRSNHSHSDVPSFDKVSPGMINEMFYHSNGVPS<br>DFQRSSLSTRNQSSLQHYGDLYADDSRYFVPFHLSNQQYPTPEDIHLSPEFHMFQANSRHFDRHVNERNSSEIDNMMRARGNSVLRD<br>NIYGEPRGMNLNPFSGENMFPPLASTRPCMRHLGSAELLANDFNMGPAHGGLHDAFESAANLSYREQAYAQCKRSPFSASSSSPTW<br>ENDYNLPPLDEARSESYNDFSHCPAMLDMLTESNRGPRASRLNSKSKMISYDRVDRFCQQELLSQFRDAKFFVIKSYSEDNVHKSIK<br>HCVWASTKNGNKKLDAAYREAKKKDVACPVFLLFSVNRLFFFITNQNLLEALSVQTDTLLGRYIHLLVYSTMKLHCQAFVFLD
	AtECT10	AT5G58190	MDPASSSSSNQRNRIVSSGERPVMADNGVQQVSHDDGRNIVSSSYSSSVVPSPDPAPYYNVLQTPTNLYHYDWDIANSGYAQGINT<br>WDGYPRYATTTPEAMHVPPVVYNDNSSLMYQYPGYGFNPYPSVMLEGQIPVSPAYYPQPFGAPSAMHYLPSDIDPTSAAYMIPYGQ<br>YGGGNYSGNQGDISLTSHIPYPQTMGILGPYDHNASQVALHGSGVASSSSLGGYYHVGSYQNPSSTPSYYGVDNRVRLTPDIGKRRE<br>KDQGSISSTSDLYGNRGPRASSRVKSKNSSKPCSTIGDSASDSSTAGPNPSLYNHPEFVTDYKNAKFFIVKSFSEDNVHRSIKYNVWA<br>STPHGNKKLDTAYRDAEKMGGKCPIFLFFSVNASGQFCGVSEMVGPVDFEKDAGYWQQDRWSGQFPVKWHIVKDIPNNRFCHILL<br>QNNDNKPVTHSRDSQEVKLRQGIEMLRIFKEYEAHTSILDDFGYYDELEGQKVGEDGTRKKAGEEETSVEQLSERLQAVKVEDGKE<br>EEKKELIAD
	AtECT11	AT1G09810	MATEKNTLDSEATFAQTVPALDSTTKKQDESPPKSTPSLTVTACANGSDVVSSQPNDNGQAHTTDFRKGNHRDKNSSDVYADSTLR<br>GDRPKGSNCLSQTSFSAPKPLGNFNGAGRLPPNTQAHAFRPPFKGKEAAGQFLTFSNQKTSCVPYSGYINGNSNNGFWDQRDHNKK<br>PERNGESDYLVELKCGPRANAKTRPPSESSPLKQNNSFALALRREMYNLPDFQTDYEDAKFFVIKSYSEDDVHKSIKYSVWSSTING<br>NKKLDAAFRDAETKTLEDGKKRPIFLFFSVNASRQFVGLAEMVGYVDFNKDLDFWQVDKWSGFFPVEWHVVKDIPNWELRHIILD<br>NNEDKPVTHTRDTHEIKLKEGLQMLSIFKKYSAVTFLLDDMDFYEEREKSLRAKKEHKPATLRMDLFKEKDYDYEMEGNRRMNHQ<br>ERGYNWNRSSNSKTQASLVNQTKYLSIRGYSGYKKNTGNST
	AtECT12	AT4G11970	MSSDTAKENASVVDSSLTDWKQDLGNSDDPESTSYRSKEDHKLSKVDVDRRNFPDQLESAKANKNSKPGYRTRYFIIKSLNYDNIQ<br>VSVEKGIWATQVMNEPILEGAFHKSGRVILIFSVNMSGFFQGYAEMLSPVGWRRDQIWSQGGGKNNPWGRSFKVKWLRLSELPFQ<br>KTLHLKNPLNDYKPVKISRDCQELPEDIGEALCELLDANSCDDGLLNSSSRDDYSTKRSRAEPPSSSGDDEYNNNLWGHTPMSYPPT<br>VYPNQDDLSRFHLAQQRGYGVSPEYLHTSPGASNSRTEQDKSLRFNSWCLPLESPLANSLTDDDFLEMVVAYLLVTYAENFIPLRPL<br>VLELDIFCKSCCLVESRIVMTHYWKLGENICSIFQTLSYFSVLFLIHNTYISQCLAEFCIDVTGTSFCFLRSIMRSGYVVVVCLTKSCDC<br>VFLLFFAVL
	AtCPSF30-L	At1g30460	MEDADGLSFDFEGGLDSGPVQNTASVPVAPPENSSSAAVNVAPTYDHSSATVAGAGRGRSFRQTVCRHWLRGLCMKGDACGFLH<br>QFDKARMPICRFFRLYGECREQDCVYKHTNEDIKECNMYKLGFCPNGPDCRYRHAKLPGPPPPVEEVLQKIQQLTTYNYGTNRLYQ<br>ARNVAPQLQDRPQGQVPMQGQPQESGNLQQQQQQQPQQSQHQVSQTLIPNPADQTNRTSHPLPQGVNRYFVVKSNNRENFELSVQ<br>QGVWATQRSNEAKLNEAFDSVENVILIFSVNRTRHFQGCAKMTSRIGGYIGGGNWKHEHGTAQYGRNFSVKWLKLCELSFHKTRN<br>LRNPYNENLPVKISRDCQELEPSVGEQLASLLYLEPDSELMAISIAAEAKREEEKAKGVNPESRAENPDIVPFEDNEEEEEEEDESEEE<br>EESMAGGPQGRGRGRGIMWPPQMPLGRGIRPMPGMGGFPLGVMGPGDAFPYGPGGYNGMPDPFGMGPRPFGPYGPRFGGDFRGP<br>VPGMMFPGRPPQQFPHGGYGMMGGGRGPHMGGMGNAPRGGRPMYYPPATSSARPGPSNRKTPERSDERGVSGDQQNQDASHDM<br>EQFEVGNSLRNEESESEDEDEAPRRSRHGEGKKRR
Homo sapiens (Human)	HsMETTL3	Q86U44	MSDTWSSIQAHKKQLDSLRERLQRRRKQDSGHLDLRNPEAALSPTFRSDSPVPTAPTSGGPKPSTASAVPELATDPELEKKLLHHLSD<br>LALTLPTDAVSICLAISTPDAPATQDGVESLLQKFAAQELIEVKRGLLQDDAHPTLVTYADHSKLSAMMGAVAEKKGPGEVAGTVT<br>GQKRRAEQDSTTVAAFASSLVSGLNSSASEPAKEPAKKSRKHAASDVDLEIESLLNQQSTKEQQSKKVSQEILELLNTTTAKEQSIVE<br>KFRSRGRAQVQEFCDYGTKEECMKASDADRPCRKLHFRRIINKHTDESLGDCSFLNTCFHMDTCKYVHYEIDACMDSEAPGSKDHT<br>PSQELALTQSVGGDSSADRLFPPQWICCDIRYLDVSILGKFAVVMADPPWDIHMELPYGTLTDDEMRRLNIPVLQDDGFLFLWVTGR<br>AMELGRECLNLWGYERVDEIIWVKTNQLQRIIRTGRTGHWLNHGKEHCLVGVKGNPQGFNQGLDCDVIVAEVRSTSHKPDEIYGMI<br>ERLSPGTRKIELFGRPHNVQPNWITLGNQLDGIHLLDPDVVARFKQRYPDGIISKPKNL
	HsMETTL14	Q9HCE5	MDSRLQEIRERQKLRRQLLAQQLGAESADSIGAVLNSKDEQREIAETRETCRASYDTSAPNAKRKYLDEGETDEDKMEEYKDELEM<br>QQDEENLPYEEEIYKDSSTFLKGTQSLNPHNDYCQHFVDTGHRPQNFIRDVGLADRFEEYPKLRELIRLKDELIAKSNTPPMYLQADI

			EAFDIRELTPKFDVILLEPPLEEYYRETGITANEKCWTWDDIMKLEIDEIAAPRSFIFLWCGSGEGLDLGRVCLRKWGYRRCEDICWIKTNKNNPGKTKTLDPKAVFQRTKEHCLMGIKGTVKRSTDGDFIHANVDIDLIITEEPEIGNIEKPVEIFHIIEHFCLGRRRLHLFGRDSTIRPGWLTVGPTLTNSNYNAETYASYFSAPNSYLTGCTEEIERLRPKSPPPKSKSDRGGGAPRGGGRGGTSAGRGRERNRSNFRGERGGFRGGRGGAHRGGFPPR
	HsMETTL4	Q8N3J2	MSVVHQLSAGWLLDHLSFINKINYQLHQHHEPCCRKKEFTTSVHFESLQMDSVSSSGVCAAFIASDSSTKPENDDGGNYEMFTRKFVFRPELFDVTKPYITPAVHKECQQSNEKEDLMNGVKKEISISIIGKKRKRCVVFNQGELDAMEYHTKIRELILDGSLQLIQEGLKSGFLYPLFEKQDKGSKPITLPLDACSLSELCEMAKHLPSLNEMEHQTLQLVEEDTSVTEQDLFLRVVENNSSFTKVITLMGQKYLLPPKSSFLLSDISCMQPLLNYRKTFDVIVIDPPWQNKSVKRSNRYSYLSPLQIQQIPIPKLAAPNCLLVTWVTNRQKHLRFIKEELYPSWSVEVVAEWHWVKITNSGEFVFPLDSPHKKPYEGLILGRVQEKTALPLRNADVNVLPIPDHKLIVSVPCTLHSHKPPLAEVLKDYIKPDGEYLELFARNLQPGWTSWGNEVLKFQHVDYFIAVESGS
	HsALKBH1	Q13686	MGKMAAAVGSVATLATEPGEDAFRKLFRFYRQSRPGTADLEGVIDFSAAHAARGKGPGAQKVIKSQLNVSSVSEQNAYRAGLQPVSKWQAYGLKGYPGFIFIPNPFLPGYQWHWVKQCLKLYSQKPNVCNLDKHMSKEETQDLWEQSKEFLRYKEATKRRPRSLLEKLRWVTVGYHYNWDSKKYSADHYTPFPSDLGFLSEQVAAACGFEDFRAEAGILNYYRLDSTLGIHVDRSELDHSKPLLSFSFGQSAIFLLGGLQRDEAPTAMFMHSGDIMIMSGFSRLLNHAVPRVLPNPEGEGLPHCLEAPLPAVLPRDSMVEPCSMEDWQVCASYLKTARVNMTVRQVLATDQNFPLEPIEDEKRDISTEGFCHLDDQNSEVKRARINPDS
	HsALKBH2	Q6NS38	MDRFLVKGAQGGLLRKQEEQEPTGEEPAVLGGDKESTRKRPRREAPGNGGHSAGPSWRHIRAEGLDCSYTVLFGKAEADEIFQELEKEVEYFTGALARVQVFGKWHSVPRKQATYGDAGLTYTFSGLTLSPKPWIPVLERIRDHVSGVTGQTFNFVLINRYKDGCDHIGEHRDDERELAPGSPIASVSFGACRDFVFRHKDSRGKSPSRRVAVVRLPLAHGSLLMMNHPTNTHWYHSLPVRKKVLAPRVNLTFRKILLTKK
	HsALKBH3	Q96Q83	MEEKRRRARVQGAWAAPVKSQAIAQPATTAKSHLHQKPGQTWKNKEHHLSDREFVFKEPQQVVRRAPEPRVIDREGVYEISLSPTGVSRVCLYPGFVDVKEADWILEQLCQDVPWKQRTGIREDITYQQPRLTAWYGELPYTYSRITMEPNPHWHPVLRTLKNRIEENTGHTFNSLLCNLYRNEKDSVDWHSDDEPSLGRCPIIASLSFGATRTFEMRKKPPPEENGDYTYVERVKIPLDHGTLLIMEGATQADWQHRVPKEYHSREPRVNLTFRTVYPDPRGAPW
	HsALKBH4	Q9NXW9	MAAAAAETPEVLRECGCKGIRTCLICERQRGSDPPWELPPAKTYRFIYCSDTGWAVGTEESDFEGWAFPFPGVMLIEDFVTREEEAELVRLMDRDPWKLSQSGRRKQDYGPKVNFRKQKLKTEGFCGLPSFSREVVRRMGLYPGLEGFRPVEQCNLDYCPERGSAIDPHLDDAWLWGERLVSLNLLSPTVLSMCREAPGSLLLCSAPSAAPEALVDSVIAPSRSVLCQEVEVAIPLPARSLLVLTGAARHQWKHAIHRRHIEARRVCVTFRELSAEFGPGGRQQELGQELLRIALSFQGRPV
	HsALKBH5	Q6P6C2	MAAASGYTDLREKLKSMTSRDNYKAGSREAAAAAAAAVAAAAAAAAAAEPYPVSGAKRKYQEDSDPERSDYEEQQLQKEEEARKVKSGIRQMRLFSQDECAKIEARIDEVVSRAEKGLYNEHTVDRAPLRNKYFFGEGYTYGAQLQKRGPGQERLYPPGDVDEIPEWVHQLVIQKLVEHRVIPEGFVNSAVINDYQPGGCIVSHVDPIHIFERPIVSVSFFSDSALCFGCKFQFKPIRVSEPVLSLPVRRGSVTVLSGYAADEITHCIRPQDIKERRAVIILRKTRLDAPRLETKSLSSSVLPPSYASDRLSGNNRDPALKPKRSHRKADPDAAHRPRILEMDKEENRRSVLLPTHRRRGSFSSENYWRKSYESSEDCSEAAGSPARKVKMRRH
	HsALKBH6	Q3KRA9	MEEQDARVPALEPFRVEQAPPVIYYVPDFISKEEEEYLLRQVFNAPKPKWTQLSGRKLQNWGGLPHPRGMVPERLPPWLQRYVDKVSNLSLFGGLPANHVLVNQYLPGEGIMPHEDGPLYYPTVSTISLGSHTVLDFYEPRRPEDDDPTEQPRPPPRPTTSLLLEPRSLLVLRGPAYTRLLHGIAAARVDALDAASSPPNAAACPSARPGACLVRGTRVSLTIRRVPRVLRAGLLLGK
	HsALKBH7	Q9BT30	MAGTGLLALRTLPGPSWVRGSGPSVLSRLQDAAVVRPGFLSTAEEETLSRELEPELRRRRYEYDHWDAAIHGFRETEKSRWSEASRAILQRVQAAAFGPGQTLLSSVHVLDLEARGYIKPHVDSIKFCGATIAGLSLLSPSVMRLVHTQEPGEWLELLLEPGSLYILRGSARYDFSHEILRDEESFFGERRIPRGRRISVICRSLPEGMGPGESGQPPPAC
	HsALKBH8	Q96BT7	MDSNHQSNYKLSKTEKKFLRKQIKAKHTLLRHEGIETVSYATQSLVVANGGLGNGVSRNQLLPVLEKCGLVDALLMPPNKPYSFARYRTTEESKRAYVTLNGKEVVDDLGQKITLYLNFVEKVQWKELRPQALPPGLMVVEEIISSEEEKMLLESVDWTEDTDNQNSQKSLKHRRVKHFGYEFHYENNNVDKDKPLSGGLPDICESFLEKWLRKGYIKHKPDQMTINQYEPGQGIPAHIDTHSAFEDEIVSLSLGSEIVMDFKHPDGIAVPVMLPRRSLLVMTGESRYLWTHGITCRKFDTVQASESLKSGIITSDVGDLTLSKRGLRTSFTFRKVRQTPCNCSYPLVCDSQRKETPPSFPESDKEASRLEQEYVHQVYEEIAGHFSSTRHTPWPHIVEFLKALPSGSIVADIGCGNGKYLGINKELYMIGCDRSQNLVDICRERQFQAFVCDALAVPVRSGSCDACISIAVIHHFATAERRVAALQEIVRLLRPGGKALIYVWAMEQEYNKQKSKYLRGNRNSQGKKEEMNSDTSVQRSLVEQMRDMGSRDSASSVPRINDSQEGGCNSRQVSNSKLPVHVNRTSFYSQDVLVPWHLKGNPDKGKPVEPFGPIGSQDPSPVFHRYYHVFREGELEGACRTVSDVRILQSYYDQGNWCVILQKA
	HsFTO	Q9C0B1	MKRTPTAEEREREAKKLRLLEELEDTWLPYLTPKDDEFYQQWQLKYPKLILREASSVSEELHKEVQEAFLTLHKHGCLFRDLVRIQGKDLLTPVSRILIGNPGCTYKYLNTRLFTVPWPVKGSNIKHTEAEIAAACETFLKLNDYLQIETIQALEELAAKEKANEDAVPLCMSADFPRVGMGSSYNGQDEVDIKSRAAYNVTLLNFMDPQKMPYLKEEPYFGMGKMAVSWHHDENLVDRSAVAVYSYSCEGPEEESEDD

			SHLEGRDPDIWHVGFKISWDIETPGLAIPLHQGDCYFMLDDLNATHQHCVLAGSQPRFSSTHRVAECSTGTLDYILQRCQLALQNVC<br>DDVDNDDVSLKSFEPAVLKQGEEIHNEVEFEWLRQFWFQGNRYRKCTDWWCQPMAQLEALWKKMEGVTNAVLHEVKREGLPVE<br>QRNEILTAILASLTARQNLRREWHARCQSRIARTLPADQKPECRPYWEKDDASMPLPFDLTDIVSELRGQLLEAKP
	HsYTHDF1	Q9BYJ9	MSATSVDTQRTKGQDNKVQNGSLHQKDTVHDNDFEPYLTGQSNQSNSYPSMSDPYLSSYYPPSIGFPYSLNEAPWSTAGDPPIPYLT<br>TYGQLSNGDHHFMHDAVFGQPGGLGNNIYQHRFNFFPENPAFSAWGTSGSQGQQTQSSAYGSSYTYPPSSLGGTVVDGQPGFHSDT<br>LSKAPGMNSLEQGMVGLKIGDVSSSAVKTVGSVVSSVALTGVLSGNGGTNVNMPVSKPTSWAAIASKPAKPQPKMKTKSGPVMG<br>GGLPPPPIKHNMDIGTWDNKGPVPKAPVPQQAPSPQAAPQPQQVAQPLPAQPPALAQPQYQSPQQPPQTRWVAPRNRNAAFGQSGG<br>AGSDSNSPGNVQPNSAPSVESHPVLEKLKAAHSYNPKEFEWNLKSGRVFIIKSYSEDDIHRSIKYSIWCSTEHGNKRLDSAFRCMSSK<br>GPVYLLFSVNGSGHFCGVAEMKSPVDYGTSAGVWSQDKWKGKFDVQWIFVKDVPNNQLRHIRLENNDNKPVTNSRDTQEVPLEK<br>AKQVLKIISSYKHTTSIFDDFAHYEKRQEEEEVVRKERQSRNKQ
	HsYTHDF2	Q9Y5A9	MSASSLLEQRPKGQGNKVQNGSVHQKDGLNDDDFEPYLSPQARPNNAYTAMSDSYLPSYYSPSIGFSYSLGEAAWSTGGDTAMPY<br>LTSYGQLSNGEPHFLPDAMFGQPGALGSTPFLGQHGFNFFPSGIDFSAWGNNSSQGQSTQSSGYSSNYAYAPSSLGGAMIDGQSAFA<br>NETLNKAPGMNTIDQGMAALKLGSTEVASNVPKVVGSAVGSGSITSNIVASNSLPPATIAPPKPASWADIASKPAKQQPKLKTKNGI<br>AGSSLPPPPIKHNMDIGTWDNKGPVAKAPSQALVQNIGQPTQGSPQPVGQQANNSPPVAQASVGQQTQPLPPPPPQPAQLSVQQQA<br>AQPTRWVAPRNRGSGFGHNGVDGNGVGQSQAGSGSTPSEPHPVLEKLRSINNYNPKDFDWNLKHGRVFIIKSYSEDDIHRSIKYNIW<br>CSTEHGNKRLDAAYRSMNGKGPVYLLFSVNGSGHFCGVAEMKSAVDYNTCAGVWSQDKWKGRFDVRWIFVKDVPNSQLRHIRL<br>ENNENKPVTNSRDTQEVPLEKAKQVLKIIASYKHTTSIFDDFSHYEKRQEEEESVKKERQGRGK
	HsYTHDF3	Q7Z739	MSATSVDQRPKGQGNKVSVQNGSIHQKDAVNDDDFEPYLSSQTNQSNSYPPMSDPYMPSYYAPSIGFPYSLGEAAWSTAGDQPMP<br>YLTTYGQMSNGEHHYIPDGVFSQPGALGNTPPFLGQHGFNFFPGNADFSTWGTSGSQGQSTQSSAYSSSYGYPPSSLGRAITDGQAG<br>FGNDTLSKVPGISSIEQGMTGLKIGGDLTAAVTKTVGTALSSSGMTSIATNSVPPVSSAAPKPTSWAAIARKPAKPQPKLKPKGNVGI<br>GGSAVPPPPIKHNMNIGTWDEKGSVVKAPPTQPVLPPQTIIQQPQPLIQPPPLVQSQLPQQQPQPPQPQQQQGPQPQAQPHQVQPQQQ<br>QLQNRWVAPRNRGAGFNQNNGAGSENFGLGVVPVSASPSSVEVHPVLEKLKAINNYNPKDFDWNLKNGRVFIIKSYSEDDIHRSIK<br>YSIWCSTEHGNKRLDAAYRSLNGKGPLYLLFSVNGSGHFCGVAEMKSVVDYNAYAGVWSQDKWKGKFEVKWIFVKDVPNNQLR<br>HIRLENNDNKPVTNSRDTQEVPLEKAKQVLKIIATFKHTTSIFDDFAHYEKRQEEEEAMRRERNRNKQ
	HsYTHDC1	Q96MU7	MAADSREEKDGELNVLDDILTEVPEQDDELYNPESEQDKNEKKGSKRKSDRMESTDTKRQKPSVHSRQLVSKPLSSSVSNNKRIVST<br>KGKSATEYKNEEYQRSERNKRLDADRKIRLSSSASREPYKNQPEKTCVRKRDPERRAKSPTPDGSERIGLEVDRRASRSSQSSKEEVN<br>SEEYGSDHETGSSGSSDEQGNNTENEEEGVEEDVEEDEEVEEDAEEDEEVDEDGEEEEEEEEEEEEEEEEEEEEYEQDERDQKEEGN<br>DYDTRSEASDSGSESVSFTDGSVRSGSGTDGSDEKKKERKRARGISPIVFDRSGSSASESYAGSEKKHEKLSSSVRAVRKDQTSKLKY<br>VLQDARFFLIKSNNHENVSLAKAKGVWSTLPVNEKKLNLAFRSARSVILIFSVRESGKFQGFARLSSESHHGGSPIHWVLPAGMSAK<br>MLGGVFKIDWICRRELPFTKSAHLTNPWNEHKPVKIGRDGQEIELECGTQLCLLFPPDESIDLYQVIHKMRHKRRMHSQPRSRGRPSR<br>REPVRDVGRRRPEDYDIHNSRKKPRIDYPPEFHQRPGYLKDPRYQEVDRRFSGVRRDVFLNGSYNDYVREFHNMGPPPPWQGMPPY<br>PGMEQPPHHPYYQHHAPPPQAHPPYSGHHPVPHEARYRDKRVHDYDMRVDDFLRRTQAVVSGRRSRPRERDRERERDRPRDNRR<br>DRERDRGRDRERERERLCDRDRDRGERGRYRR
	HsYTHDC2	Q9H6S0	MSRPSSVSPRQPAPGGGGGGGPSPCGPGGGGRAKGLKDIRIDEEVKIAVNIALERFRYGDQREMEFPSSLTSTERAFIHRLSQSLGLVS<br>KSKGKGANRYLTVKKKDGSETAHAMMTCNLTHNTKHAVRSLIQRFPVTNKERTELLPKTERGNVFAVEAENREMSKTSGRLNNGI<br>PQIPVKRGESEFDSFRQSLPVFEKQEEIVKIIKENKVVLIVGETGSGKTTQIPQFLLDDCFKNGIPCRIFCTQPRRLAAIAVAERVAAERR<br>ERIGQTIGYQIRLESRVSPKTLLTFCTNGVLLRTLMAGDSTLSTVTHVIVDEVHERDRFSDFLLTKLRDLLQKHPTLKLILSSAALDVN<br>LFIRYFGSCPVIYIQGRPFEVKEMFLEDILRTTGYTNKEMLKYKKEKQQEEKQQTTLTEWYSAQENSFKPESQRQRTVLNVTDEYDL<br>LDDGGDAVFSQLTEKDVNCLEPWLIKEMDACLSDIWLHKDIDAFAQVFHLILTENVSVDYRHSETSATALMVAAGRGFASQVEQLI<br>SMGANVHSKASNGWMALDWAKHFGQTEIVDLLESYSATLEFGNLDESSLVQTNGSDLSAEDRELLKAYHHSFDDEKVDLDLIMHL<br>LYNICHSCDAGAVLIFLPGYDEIVGLRDRILFDDKRFADSTHRYQVFMLHSNMQTSDQKKVLKNPPAGVRKIILSTNIAETSITVNDV<br>VFVIDSGKVKEKSFDALNFVTMLKMVWISKASAIQRKGRAGRCRPGICFRLFSRLRFQNMLEFQTPELLRMPLQELCLHTKLLAPVN<br>CPIADFLMKAPEPPPALIVRNAVQMLKTIDAMDTWEDLTELGYHLADLPVEPHLGKMVLCAVVLKCLDPILTIACTLAYRDPFVLPT<br>QASQKRAAMLCRKRFTAGAFSDHMALLRAFQAWQKARSDGWERAFCEKNFLSQATMEIIIGMRTQLLGQLRASGFVRARGGGDIR<br>DVNTNSENWAVVKAALVAGMYPNLVHVDRENLVLTGPKEKKVRFHPASVLSQPQYKKIPPANGQAAAIKALPTDWLIYDEMTRA<br>HRIANIRCCSAVTPVTILVFCGPARLASNALQEPSSFRVDGIPNDSSDSEMEDKTTANLAALKLDEWLHFTLEPEAASLLLQLRQKWH<br>SLFLRRMRAPSKPWSQVDEATIRAIIAVLSTEEQSAGLQQPSGIGQRPRPMSSEELPLASSWRSNNSRKSSADTEFSDECTTAERVLMK<br>SPSPALHPPQKYKDRGILHPKRGTEDRSDQSSLKSTDSSSYPSPCASPSPPSSGKGSKSPSPRPNMPVRYFIMKSSNLRNLEISQQKGIW<br>STTPSNERKLNRAFWESSIVYLVFSVQGSGHFQGFSRMSSEIGREKSQDWGSAGLGGVFKVEWIRKESLPFQFAHHLLNPWNDNKK<br>VQISRDGQELEPLVGEQLLQLWERLPLGEKNTTD

Phaseolus vulgaris	PvMTA	Phvul.010G 102500.1	METQSDGNEDTIAAIKDMRQQLEARIECQHKAHMEMLASIQAVIPNLVSSLDLSLRVVSSFNQRPFAPTPALPLPDPKLNPKKPIELT<br>HRSNSESYADGSTEVDLTNPRNQKLKTSIDSNPASQVDSEKVSPLAVVRSLVAVCLLGRVPFSPIDSSTVSRKLENDQAVTPAEKSAL<br>QELGGDSGAILAVEIALRSMADDNGGVEVEEFVVSGKARIMVLNIDRTRLLRELPESAQYQQLESSSGDGNVNQNQVQQITNNGTN<br>VNGGLLGMGRPVLRPMSEMWIPHGDPHMSGLQPMFSGGPRGAPRVMGMMGTHRGMSIPSMHRLPLGPNAQGSSPNAMSQKPRTL<br>EDDMKDLEALLNKKSFRELQKSKTGEELLDLIHRPTARETAVAAKFKTKGGSQVRQYCDLLTKEDCRRQTGSFIACDKVHFRRIIAP<br>HTDINLGDCSFLDTCRHMKTCKYVHYEYDPTPDVSPTMMGAPPPPKPLKPQRAEYCSEVELGEPQWINCDIRNFRMDILGQFGVIM<br>ADPPWDIHMELPYGTMADDEMRSLNVPALQTDGLIFLWVTGRAMELGRECLELWGYKRVEEIIWVKTNQLQRIIRTGRTGHWLNH<br>SKEHCLVGIKGNPEVNRNIDTDVIVAEVRETSRKPDEMYPMLERISPRTRKLELFARMHNTHAGWMSLGNQLSGVRLVDEGLRARF<br>KAAYPDVEVQPPSPPRPSAMEVDTGVAAHTRSPFAAAESKSNSTQFAETAAAPETSFASEDKSMAIDVDIG
	PvMTB	Phvul.007G 073300.1	MDSSDSGRGYSKRERDDEDWEFSDKRKDRSRKFGSNGDEGEGSDGGARRKRSSRTDSDDYDSRSKGAKKRQEESTLEKLSSWYED<br>GELDDKSARKRAMDGDFHESVVSKEDGKGDGGGGGREKVGHESRSRSSRRKWDEVDASSVRRSQDEKGEFRSGKRDSSRDRERSGS<br>ARSEHGEGKASGADRVVKSSSKEDRRGDSERGKSKGKSDSVDAGREERVEKPRHHRALGSDGAETWDRSLNAEEDGHVRVRDKS<br>ARESGNSNRSRTPERSGKRHQDLENSEVDYERSGSFKRKEHEGDGFKDDRSKGKDDAWNDRRKDRESSKESWKRRQPSNADKEK<br>NEEGAFDDNRDWELPRHGYERMDNERPHGRFGGRKDVSRGEAVKTSTKFGISNDNYDVIEIQTKFYDYGKSESMSNHTKRNEAHQ<br>QYNAKSGVNDEEWPYHQEERGRKNDVSGDDLKERYTDDDYDFYGGRGRGQKGGVSARSTGGQSSGSGGSQPQYGNPESGSFNRA<br>GPQGMKGNRVGRGGRIRPTGRDNQQVGMPLPMMGSPYGPLAMPPPGPMQPLSHGMSPAPGPPMSPGVFLSPFTPAVWPGARGVD<br>MNIIGVPPVSPVPPGPSGPRFNASNLGNPPNPAMYYNQSGPGRGMPPNISTSGFNPPGSMGRGAPPDKSPGGWAPPKSSGALGKAPS<br>RGEQNDYSQNFVDTGMRPQNFIRELELTNVVEDYPKLRELIQKKDEIVEKSASAPLYYKCDLKEFELSPEFFGTKFDVILVDPPWEEY<br>VHRAPGVADHMEYWTFEEIMNLKIEAIADTPSFIFLWVGDGVGLEQGRQCLKKWGFRRCEDICWVKTNKSNATPGLRHDSHTLFQ<br>HSKEHCLMGIKGTVRRSTDGHIIHANIDTDVIIAEEPPYGSTQKPEDMYRIIEHFALGRRRLELFGEDHNIRAGWLTAGKELSSSNFNK<br>EAYVKNFSDKDGKVWQGGGGRNPPPEAPHLVVTTSDIEALRPKSPMKNQQQMQQQNSVSISLTTGSGSNRRPAGNSPQNPPALSVN<br>QDASSSNPSTPAPWGSPLEGFKGREGSVLPSDDKVMDIYGFHGPTPAGYLDFESYRQMNML
	PvMTC	Phvul.001G 016200.1	MEGEHNSHKLSSFYESGVYSFDDSNAVFVDSVRVLNRFYHRFSVSPSTYYSRFFKTQTTPNSVSSTVTSSLRKRKRRRESRPLNERE<br>LIALQRHQEARPLLLAAHECLLKSTEVLNALKALKSESSCSTRECDGGQRSFVDLGHVAPQLEVTLSLRVSDTDADPHPLPKDLDDF<br>PSGQCVQKVLRAFNNVVANDTQDDAVAEILSNPYIMPRESCFYMSDLGQIRNLIPAHADAGFNLIMVDPPWENASAHQKSRYQTLP<br>NRYFLSLPIKQLTHTEGALVALWVTNREKLRSFIESELFPAWGVSYAATFYWLKVKENGSLICDLDLFHHRPYECLILGYSPGKVNN<br>TDNLSEFKPVKNDRVIMSIPGDYSRKPPIADLLVEHVPGLKPPRCIELFAREILAGWVAWGNEPLYFQDSKYFVKKTVK
	PvALKBH1A	Phvul.001G 262100.1	MYVSENNRDDSDRTAFRKAEKKYKLYYDNNASSKNKKKKQPKPVDLTEVLDFRSILECYLRNGALPPGVIVLHENFTSPVFSLQNR<br>PGFYFIPGALTMEKQCSLIRESLTDFPQPPNRTNHNAMYGPIQDVFGAAKEGKVLVEDNSPNSSSETDADVDHGDCKEWKFATQKE<br>VLLRKCKSVSASTLLRKLRWSTLGLQFDWSKRNYDMSLPHNKIPEALCELAKQLAKSALPAGVEFNPEAAIVNYFGLGDTLGGHLD<br>DMEADWSKPIVSLSLGCKAIFLLGGKSREDSPLAMFLRSGDVVLMAGDARECFHGVPRIFTDEENAEIGHLEKQLTHEEDLCFLQYI<br>QTSRININIRQVF*
	PvALKBH1B	Phvul.009G 262600.1	MERSGSGRGGGRGRGRGRGGGRGRRSPSTPPVVVGKEQCGCVRGSEEKNRPQNSSPDSHSDSDSVGIGVAKLKICDDVDKPSPPPP<br>AAAAVGEGVFDICPRKQPGTVVLKPSLLVKNRERRRSSNSSSLLLRPGMVLLRGYLSLSDQKKIVERCRELGVGVGGFYQPGYGEG<br>VEMHLKMMCLGKNWDPHTSQYGEQRPFDGAKPPLIPPEFHTLVSNALRDSNALFPHDPLPSISPDICIVNFYSQTGRLGLHQDKDES<br>KDSLRRGLPVVSFSIGYSAQFLYGDHRDPDKAQKLQLESGDVLIFGGPSRNVFHGVTAIDTTTAPNLLQHINLRPGRLNLTFRQY*
	PvALKBH1C	Phvul.001G 131400.1	MGKMFKSTTKSSPPVADSAVPDSAVRKDHVVADHDTDKCIMIGSIPVLLSKKSPSCNSIGDKQHPESMSNSNHSISSSRNATNVFNPR<br>NSKRKTRVHLESAFSGEKYSVPSSNFEISKLPLPNKVSETNLSTPQDESLQSSKYWWKKPDSFNRPHNSNTNSNYAAVGAPKLNSSV<br>NKLQFRPYDICTHGRRNHVLIGATLPGENKEGRFEMQEEKTEGFVLRPGMVLLKNFISHDDQVGIVKVCRKLGLGPGGFYQPGYAN<br>GAKLRLKMMCLGMDWDPQTYKYGTKRAFDDSTPPSIPNQFSELVARSIQKAQSLTKEEYRVHDVNDVLPSMTPDICIVNFYTNYGK<br>LGLHQDRDESKESLRKGLPVVSFSIGDSADFLYGDERNEEEAEAVILDSGDVLIFGGESRHVFHGVPSVLPKSAPKQLLRDSNLSPGR<br>LNLTFRQY*
	PvALKBH2A	Phvul.006G 137400.1	MNVLKLKAVPEENPKESVKRETVDLGNGSDIVYIQRLIPSDQSWKWFQYLDKHIPWTRPTIRVFGKSFLQPRDTCYVATPGLTELTY<br>SGYQPHAYSWDDYPPLKDILDAVHKALPGSSFNSLLLNRYNGGNDYVGWHSDDEKLYGPTPEIASLTFGCERDFVLKKKPCKKSCD<br>GSDEPASKRLKKGSHDDQHTFRLKHGSLLVMRGYTQRDWIHSVPKRTKAEATRINLTFRRVF*
	PvALKBH2B	Phvul.006G 137611.1	MNVLKLKAVPEENPKESVKRETVDLGNGSDVVYIQRLIPSDQSWKWFQYLDKHIPWTRPTIRVFGKSFLQPRDTCYVATPGLTELTY<br>SGYQPHAYSWDDYPPLKDMLDAVHKALPGSSFNSLLLNRYNGGNDYVGWHSDDEKLYGPTPEIASLSLGCERDFVLKKKPCKKSC<br>DGSDEPASKRLKKGSHDDQHTFRLKHGSLLVMRGYTQRDWIHSVPKRTKAEATRINLTFRRVF*
	PvALKBH6	Phvul.004G 131600.1	MEEKENLGQYKVGSLPTLFYVPDFITDSDQSLLLNNIYEAPASKWKMLKNRRLQNWGGVVHEKGLLPQVLPPWLTNLTQKIYDES<br>ALFPSPMNHVLINEYQPNQGIMPHQDGPAYFPVVAILSLGSPVVMDFTPHARFKQDSQDDIDKDSDLEIGKDKWLDDHHPFTVLLMP

			RSLLIFKDKAYSDYLHGIQDCMLHCYNGAVNETQALKHKESDGDFFNSEDALDTIGKEEYKNISRTANRVSLTCRLVPKVHKKLFRF*
	PvALKBH7	Phvul.008G264300.1	MKEEEEKTVLEQVFGSSSSDESDSSDGYSDGEREWDSISEVKGLWLCSNFLSPRRQSHILASIQSQNWFPSPSVNQAMRFGLQHLPSWAPPLAHSIRRSIRRQAHNPPPFPPHLLHREPLFDQMIANVYQPGEGICAHVDLLRFDDGIAILSLESDCVMHFTNASLSVPVLLTPGSLILMSGEARYRWKHEINRSPEFQIWQGRHLTQSKRTSITLRKLSPSTP*
	PvALKBH8	Phvul.002G123600.1	MGLPRFGRTKNDGELSSNLYVANCGPAVGISDDDIASVFCKFGELKGVYAADESGTRVIVAYAEEGSAQAALKALHGRPCPELGGRSMHIRYSVLQPTTQDQATDLVPVSITASEVSISGLYLIHDFISAKEEEELLQAVDCRPWNSLAKRRVQHYGYEFRYDTRNVNTRHCLGELPSFVSPILERISSCPSFKNIKNIVLDQLTVNEYPPGVGLSPHIDTHSAFEDLIFSLSLSGPCIMEFRRYENGDWLPKVASSSVAKIENTEDQSNFIRRTIYLPPRSLLLLSGEARYAWHHYIPHHKIDKVNGSVIRRASRRVSFTLRKVRAGLCKCEFSQYCDSQR*
	PvALKBH9A	Phvul.001G044000.2	MEEQHHRQPKPNSINASESDPFLLNYTPSDLRTASEFLATWLPFLSRDLCTRCTQSLSDRIQSIDPGEVPQNDSPNEQIDAEDNCDAHSLGSWKDGAEVNTSVETPSQRICWADMAQEDDEFGEEENSNNGVTVDVGDSNDTSDVGTVVAEKPTLPREQREYIRFMNVRRKKDFICFERVNGKLVNILEGLELHTGIFSAAEQKRIVSYVASLQEMGKRGELKERTFSAPQKWMRGKGRQTIQFGCCYNYAVDRDGNPPGILAHSKVDPIPDLFKVIIRRLIKWHVLPPTCVPDSCIVNIYEEGDCIPPHIDNHDFVRPFCTVSFLSECNIVFGSNLKIVGPGEFDGSIAIPLPMGSVLVLNGNGADVAKHCVPAVPSRRISITFRRMDESRRPFGYVPEPDLQGIQPLAYEEVEQEKKSGGHRGGRHMRRHRDRRGGGRNDAMGFASRNDRFSEHRDSNHSTPRYGNRWSRKPGS*
	PvALKBH9B	Phvul.006G214800.1	MIYALSMEFLRSLKKDDILDLFSHDFCSKCQHLLHSRITTLRKRKLDETWSTDAGFEYSLDMPLNGSASNSQSLTGKQWDNPRNSSQRRDSATSVNKCLVGCKQSDFELLLNDGICEDSSFENGLSEEKKEKIRYSLVCCRKDFTFVEKVNGRRINVVQGLELHTEVFNALEQKKIVEWMYRLQWRGKEGKLKDRTYSEPRKWMRGKGRVTIQFGCCYNYAVDRSGNPPGIMRDEEVDPLPPVFKQIIKRMIRWNIVPSSCIPDSCIVNIYEEGDCIPPHIDHRDFVRPFYTVSFLSECKILFGSNLQIVSAGEFAGPVSISLPVGSVFVLKGHGADTAKHCIPSVSSKRISITFRKMKQSKLPYKFSPDPDLVGIKPLSFSSLNKSDKAQDEDRNLNIQQHKAESVESESDVVCNTKKASFRVK*
	PvALKBH10A	Phvul.001G147800.1	MAAVPVSRADPPAMVPPPLLVSDSFAKDAILAWFRGEFAAANAIIDTLCGHLAHLATASSEYEATFAAIHRRRMNWIPVIQMQKYHSIADIALELRRVADRKTETEGARKSESSLDDEQKLEKETLETAGNEADEAAPEYDSPDSEITDSGSQEMQPSALNNNICSNHEECEGRSSQIKLTKGFSAKESVKGHMVNVVKGLKLYEDVFSESEICKLTDFVNDIHAAGQNGELSGETFILFNKQMKGNKRELIQLGVPIFGQIKDDTKNNIEPIPALVHDVIDHLIQWKLIPEYKRPNGCIINFFEEGEFSQPFLKPPHLDQPLSTLLLSESTMAFGRILVSENDGNYKGPLMLSLKEGSLIVMRGNSADMSRHVMCPSPNRRVSISFFRVKPDSNQCQSPNPTMTAAMTLWQPGITSPYTLPNGALSGYEVMDMMPKWGIFGAPMVMLPPMRPMAVHSRKLPRGGTGVFLPWKGASRKHARHLPPRAQKGRLMELPSPVESHLGESTSEPSIAVEG*
	PvALKBH10B	Phvul.007G168900.1	MATGPTAPSDRPTMVPPPMLVSDSFAKDAILAWFRGEFAAANAIIDALCAHLATSAHDYDAVFAAIHRRRLNWIPVLQMQKYHSIADVTLELQRQSDRNHNAAAEDKSNEKTTSPESVVGNDAADEHEYESPQSEITDSGSQEMQANPMNVNICSNHEDCEGRSSQFKLTKGFTAKESVKGHMVNVVKGLKLYEDIFTDSELCKLTDFVNEIHVAGQNGELSGETFILFNKQMKGNKRELIQLGVPIFGQIKEDVKSNIEPIPALLQGVIDHLIQWQLLPEYKRPNGCIINFFEEGEFSQPFLKPPHLDQPVSTLLLSESTMAFGRILMSENDGNYKGPLMLSLKQGSLLVMRGNSADMARHVMCPSPNRRVSIAFFRVRPDSNQCQSPTPAMANAMTLWQPSFASPYALSNGAISSYEGMNMMPKWGVLRGPMVMLTPMRHVALNSRKLAGGGTGVFLPWNVPSRKHAKHLPPRAQKGRLLTLPSPVESQVGESTSKPNICVEG*
	PvALKBH10C1	Phvul.002G181800.1	MAMPSGNVVIQDKMQFPNGGGGAGVGEIQQHHYRQQWFVDERDGLIGWLRSEFAAANAIIDSLCHHLRVVGDPGEYDMVIGAIQQRRCNWNQVLLMQQYFSVADVTYTLQQVAWRKQQRPLDPVKVGAKEVRKPGPGYRYGHRFEPSKEGYNSSVESYSHDGNATFTRGMEKGTPTVDKSEEHKSGSKVEKVGDKGLASPEEKKDAIIKHQTDGNLKSTGSSEGYLSNLESEAVVVNDEFISNSKGNDSDSVESQHQSQSFSTIAKTFIGNEMIDGKMVNLADGLKLYEDIFDSTEVSNLVSLVNDLRISGKKGQLQGNQAYVVSRRPMKGHGREMIQLGVPIADAPVEGENMTGASKVMNVEPIPSLFEDIIERMVSSQVMTTKPDCCIVDFYNEGDHSQPHSWPSWFGRPVYTLFLTECEMTFGRLIASEHPGDYRGSLKLSLVPGSLLAMQGKSCDFAKHALPSIRKQRILVTFTKSQPKKSVPSDAQRLYLPAASSQWGPPPSRSPNHVRHSVGSKHYAALPTTGVLPAPPIRPQIPAQVGMQPLFVAAPVVPPMPYPAPVSIPPGSAGWTTAPPPRHPPPRIPAPGTGVFLPPPGSGNSQQQLPAGTLAEVNPSIETPTTMQEKENGKSNDDNSSSTSPKGKVQKQECNGHTDGTRDEAALESRED*
	PvALKBH10C2	Phvul.003G014200.1	MAMPSGNGGMPEKLQFPVGGGAASGGGEIQYRHQQWFVDERDGFIGWLRSEFAAANAIIDSLCQHLRVVGEPGVYDMVVGAIQQRRCNWTQVLLMQQYFSVSEVVYALQQVAWRRQQRFVDPAKAGSKEFRKFGSGFRQGQHRNEASKEGYNNSRNEAAKEGYNSKVESFGREMNAVVVTGGVEKGTRVIDKNGELNSGGKVGTMDNNSIASPEESKDTITNDQLDGILNGSGNFQGSLSSSECEAVGENEECTSNSKGNDSHSVQNQHQSQNASTIGKTFIGNEMFEGKMVNVVDGLKLYEDLIDSAEVSKLVSLVNDMRVAGKRGQFQGSQTFVVSKRPIKGRGREMIQLGVPIADAPPDVDNVTGLSKDKKVESIPSLFEDIIERLAASQVMTVKPDACIVDFFNEGDHSQPNSCPPWFGRPVYMLFLTECDITFGRTIVSDHPGDYRGAVKLSLVPGSLLVMQGKSTDLAKHALPSIHKQRILVTFTKSQPKTSLPNDSQRLSPAVTSHWAPPQGRTPNHMRHQLGPKHYPTIPATGVLPAPSIRAPPNGMQTLFVPTPVAPPISFASPVPIPLGSTGWASAPQRHPPPRMPVPGTGVFLPPPGSGTTSSQHLPGVVSEVNLSGETTSTGKESLKSNHNTINSSPKGKVDGNVVGRQECNGNADRSEGEEDVVGKEDESNDTTDANL*

	PvECT1	Phvul.001G<br>110200.1	MAAVAPTSDKTADLLQNLTLDSESKPIGVTEPVKKNGPGFSKGGMGKPFNPNSSFVPNGYSSAYYYGGYDGQGDWNVYSRYMNL<br>DGGMTQGVYGDNCSYMYHQGYGYMPYGAYAPPNSSSPMIQQDGQHYALQQYQYPCSYYKSPASADVSFTPNKFTATEGEISTAV<br>DADHVVSSNVTNKGNTVNMANSDFTNKNGLKSFLTSSQHTSLHSSDSYQGTSLPTTYAPLSGYQGPRMGTHGTQLPVPSDVSLISD<br>RQSKHGAKAGLSSSMVPVKDFTSQRNQGLLQQLPPFANMNGSRHPSGLELVSGFMNGMYPSNRMYGQYGNTFRANSRFGSRMGS<br>VDYKRNATGHGYGLNHFKKSMDGFSELNKGPRAAKSSDYKNIKSPGPVTLLLKGQNLPVKSDNKEVLLVPNKEQYNGKLFSENYS<br>DAKFFVIKSYSEDDIHKSIKYSVWASTPNGNKKLDAAYQEAKEKTGGCPIFLLFSVNTSGQFVGLAEMLGPVDFGKSVDYWQQDR<br>WTGCFSVKWHVIKDIPNSVMRHITLENNENKPVTNSRDTQEVKFEKGVQIVKIFKEHLSQTCILDDFGFYEAREKATQEKKSKEQQF<br>PKQISKPSDLTIGTVTLLKSLDATLMNEAATANTAEDRMNSESEGLLEGDGSTTAPEDSSKSC*
	PvECT2	Phvul.002G<br>247000.1	MATVANPADQATDLLQKLSLETQPKPLEMPEPTKKATGNQYGSVDSGNGANGGQIPSYERSVTPVLDFIDPAMCYLPNGYPSTAYYY<br>GGYDGTGNEWDDYSRYVNSEGVEMTSGVYGDNGSLVYHHGYGYAPYGPYSPAGSPVPTMGNDGQLYGPQHYQYPPYFQPLTPTS<br>APFTPTPAVLPQGEVSTSVAADQKTLPVEAANGNSNGVANGGNTKGRGPTSGYQDPRFGFDGVRSPIPWLDAPLFSDGQPRPVSSTA<br>ITPSISGGNNSASRNQTFRPNSQFMGLHHPRPMPAMGATHNFINRMYPNKLYGQYGSTVRSGMGYGTHGYDTRTNGRTWLAVDSK<br>YKTRGRSGGYFGYGNENVDGLNELNRGPRAKGGKNQKTFAPTVLAVKGQNLPATLGTDEEKDKTSTVPDRDLYNKADFPEEYAD<br>AKFFVIKSYSEDDIHKSIKYNVWASTQNGNKKLDAAYQEAQQKPGGCPVFLFFSVNTSGQFVGLAEMVGSVDFNKSVEYWQQDK<br>WNGCFPLKWHIVKDVPNNLLRHITLDNNENKPVTNSRDTQEVMLEPGLKLIKIFKEYTSKTCILDDFGFYEARQKTILEKKAKQQYP<br>KQVWEGKPTDEKVEINGEVNIQKSESELLKESNLVEKDGDDQKVAENGSVSKTGDAPKGAKPVVSESKVVLGSNGIANGC*
	PvECT3	Phvul.004G<br>080300.1	MEDLSPSSDTTKAADLLQNLSLDSEPKTAVVAEPAKKNGHGHGPARGVSKPFNLNASFTPNGHPSTAYYYGGYDGQGDWNSYSRY<br>MNFNGGMTQGVWGDGSSYLYHQGYGYTPYGAPMQHDDKFYGLQQYHYPSSYYQSPTSADGSFAANKINAQPGKISAAVSDEHTP<br>SGVINNGSSVGVVNEDSTNNNGLKDFLSSSRPSLLNSNDSTYQRAGFPAYAPLPGHQDSRVVPHGTQPALPSDALIFSDQKSNDGAKI<br>GLSSPAVPVKKTSSQRNTAIPLQLPQSMSYSSMHSSGPEPFYGFMNGIYPSNAMYNQYGNTYRANSHIGPAPYGFRTGSFDNKLKAA<br>NGRVNDHFKKNMDGFGELNKGPRSGNCSDDKSMKGPEAATLLPEGQNLLIKSDNKESLLIPNKKEYDGEDFPENYSDAKFFVIKSY<br>SEDDIHKSIKYNVWASTLNGNKKLDAAYHEAKEKPGDCPVFLLFSVNTSGQFVGLAEMVSPVDFGRTLEYWQQDRWTGCFSVKW<br>HIIKDIPNSVLRHITLENNENKPVTNSRDTQEVNFEKGIQVLKIFKEQSSKTCILDDFGFYETREKMIQERKSKEQQFPKQVSKSHDGA<br>LIDESATTGESGQKENLVEVNGSTTQAIEDCSKNC*
	PvECT5	Phvul.010G<br>165400.1	MAAIQPPQAPDRTTEEIPAEPDNMKEQGTVTIGHSRETTSQSGSLGSGGDVPLYPPNVYAPQAQAFYYRGFDNGNGEWDEYSSYVN<br>TEGLEIGSPGVYNENPSLIFHSGYGFNPQMPYGPYSPVTTPLPSVGGDTQLYSSQQFPYTGPPYYPQLIPPSLSYLNSPTPVSQPELTNL<br>VGIDQQVDNMFFGPRAGYPSVGSFGRGSFPVAPGSFGFHESQQGFEGSRSGGIWSDCSKPSERQRSLMPLSPSVSPRPMGSLGSFGPS<br>VGMASHQQQTLYGFGSGSNSYGRGYLPNQGSSFGDTSISNLNDRNCASLENSRRQGRPSASLCNCNGTLDILSEQNRGPRASKLKNQ<br>ILAEKNSVDNSKNSASIAKFQNESLNRSDFSIDYKDAKFFVIKSYSEDNVHKSIKYGVWASTPNGNRKLDAAYRQAMEKQEACPIFL<br>FFSVNASAQFCGVAEMVGPVNFDKSVDFWQQDKWSGQFPVKWHIIKDVPNSQFRHIVLENNDNKPVTNSRDTQEVKLPQGIEMLTI<br>FKNYETDVSILDDFDFYEDRQKAMQERKARQQSSMMTTGLVGENEHRSSANTTGDFMKQMSKSFALVVRLDENNNEVAADRDSL<br>VSHGPIGNVVKSDDGQSVTASPTQTS*
	PvECT6	Phvul.006G<br>121600.1	MEVYDVSETRNHDAHTIEGTDLNSHFSNPNLEQTEVMTNEGAPEFYVDQNMYYPAATNYGYYCTGFETPGEWEDHHRIFGVDGPN<br>VQYTGAQNESLPYVYYSYGYAQSPYNPYNPYIPGAMIGGDGSFGGGQHYYTLPNYQNPVSTPGYIPLVPLDNFYDSSADSFFGASAS<br>GSKPDGRGLKHKFNSASGNISRNSSKFLSNQTSSLARVSEGPRGNDGRKQDLTPASVSGGSFLNLASPAVHQSAVAKLRPKLHMGKI<br>PSGGNGSSDILGEQNRGPRVGRSKNQLSVKAYTTVTGDGNEQGNIVIYTDQYNKEDFSLDYENAKFFVIKSYSEDDVHKSIKYNVW<br>SSTPHGNKKLENAYEDAKKIAAEKSGVCPIFLFFSVNASGQFCGVAEMIGTVDFNKNMDFWQQDKWSGSFPVKWHFIKDVPNPNFR<br>HIILENNENKPVTNSRDTQEIVYLKGLEMLKIFKNHTLKTSLLDDFIYYESRQKIMLDEKAKLLGKNFDSPIFVPVLEASQKLSFTSTG<br>DYEKNLKPKDDSDGLKQILVSIPEQIASNSNVTSINPVDEKAEKTVDKDISSILKIGSVTIAPKQVEAKQSISIDNKEPVDVLTVGSMQV<br>KVNGFGSSSGFLKVGSIPLDARALQPGKGDPSVKTGSQR*
	PvECT7	Phvul.003G<br>119300.1	MEMYDVSGTRNADAYLIEGTDLNSHITNPNFEQFQVMFNDGAPEFVIDQNSYYPAATNYGYYCTGFESPREWEGHHRIFGVDGPDI<br>QYTGAQHESFPYVYYTPSYGYAQSPYNPYNPYIPGAMIGVDGSFGGAHQYYSFPNYQNPISSPSYIPFVQPDNFPNSSVDSLFDASAS<br>GSRPDGKGLKHKFSSASGAFARSSSKSLSNPTSSLARISEGPRDNAGIKKDVTSGSASGRGFLNLPLPAVRQARSIDVLTHPVDIISNG<br>NVLSHRNQLKIACPLSSEFSDCGSNANGQSAVAKLRQKALMSKGWSDVNGSSDVLGERNRGPRIGNSNSKYHQLAVKAYTNKGDG<br>NTQENIIVYTDRYNREDFPVSIEKAKYFVIKSYSEDDVHKSIKYNVWSSTPHGNKKLQNAYEDAERIAAGKSGGCPVFLFFSVNASG<br>QFCGVAEMAGPVDFNKDMDFWQQDKWSGSFPVKWHIIKDVPNANFRHIILENNENKPVTNSRDTQEIMYGKGLEMLKMFKNRSL<br>KTSLLDDFMYYENRQKIMQEEKAKMLIRNFEKNPLSLPTLEPPRKLNFVFDIPPVSKNSKTDDGLDNLKQTSRSGHIVCSSEVTNTAP<br>VDEKAEKCTVEKEDIASVFKIGSVTINPKQVETKPSAVSVSKKEADDVFTVGSMLVKVNGFAKSSTFLKIGSIPLDPRKGQLDGGTRV<br>KNG*

	PvECT8A	Phvul.005G045600.1	MAAQLQNSADEMMKKLHGDSSAAELNNSNTVPSKGASSPSDARSCVSSIGDASGSVKDVDVDHEYLSTDQGAPYPAGGYYGYYYPGYGGFFGESDNQGYYVGADAMDLQYPVMQADNGSYVYIVPGFQTGYPSYFPIGTAGVEGQYNVYHPGSIFQHPIGSPGYFPASLSYGELPPSTYSWDSSLITQDGLQGHGYNELPGKPNGRSNLSSHSHSGGIVSKSSPSSNSAEVKGSTPLLEVSSTHVKRNQPKQTNKAPVSVLHSPVAKFPTYNQGKTGFLYPNNLLSVKTNTKGWVSTDKLKNRNKVNDSLNEQNQGPRTANAKGALVSGGNSVRNLALGGSGNVTSKIRTDQYNLPDFPTKYDHALFFVIKSYSEDDIHKSIKYNVWASTPNGNKRLDGAFQDAQKRMEEKGCKCPVFLFFSVNASGQFCGVAEMTGRVDFNKSMDFWQQDKWNGYFPVKWHIIKDVPNPQLRHIILENNDHKPVTNSRDTQEVNFPQGVEILNIFKNYVARTSILDDFEFYESRQKVLQEKKTRQSMPHTSIQHVDELTTTLGSVELSSYVKNNDPKVVEKVKD*
	PvECT8B	Phvul.004G132700.1	TYVSTDKLKNKNKVNDSLNEQNQGPRTANAKGALVSGANYVRNLALGGSGNVTSKIRTDQYNLPDFPTKYDHALFFVIKSYNEEDIHKSIKYNVWASTPNGNKRLDGAYQDAQKRMEDKGCKCPVFLFFSVKWHIIKDVPNPQLRHIILENNDHKPVTNMNFPQGVEILNIFKNYVARTSILDDFEFYESRQKVLQEKKTRQPMPHTSIQVCFTLQPYSKISLNVYFSFLI*
	PvECT11	Phvul.006G218800.1	MGLLELKLVDHAAVIPQSPISRVHSFFSLCVNLITHSIHHLFSPTSLSPSNSPSIFFIMDRAQDFRKLQSVPKGTTDRSQQQNHEYVVSRKDFISSDLKSFIASSGDDAGVAGVNESEARPCYASVPTSIAVQSKTVKGYVSGKTHPKSPGSMKPNNLKSTHRNESLTSTISKTVANTEKDIKIKNKVPHLHSNFTAATAPTTYQHVGKFSSSATSRCHVFSPTNYRPNTSTDYIPNTSTNYIPNTSTNYIPNTSTNYRPNGRVSSVNDRLSLNDKFRSGESEMSKEITRGPRGHYNFFLLQPSIVNDESATTISADQYNLSDFQTEYETAKFYVIKSFNEDDVHKGVKYNVWTSTPNGNKKLNIAFLDAEAKLRQTGTKCPVFLFFSVNASRQFVGVAEMVGPVDFKKDMSFWKLDKYNGFFSIKWHIIKDVPNNQFVHIILPSNENKPVTFTRDTQEIGLKQGLEMLNIFKNYSAKTSLLDDFDFYENREKLFRSQKKAMDARPAQGVYGNANYQNTFKAREKKIEMPSRGTKQESLVTLTKNLSLNPSGKQGFR*
	PvECT12	Phvul.002G152600.1	MSSDSTKENASVVDSSVTEWRNDIGNSDDPESSCYKFNENNNPIRADIAGHSYGLVGHPTGIGVEKWNDIKYFIIKSLNRENIDLSIKKGIWATQIMNERILEEAFHNSGCVILIFSVNMSGSFQGYAQMMSSIGRGRDNVWSEGIGKSNPWGRSFKVQWLCFNDLPFYKTLHLKNPLNDYKPVKISRDCQELSPDIGLALCELLDGKNDTNDLLTSSSRDDFSFKGRYVNTPSSMGDEDCNFPPYPMSWSMPLPYSSMFYQNQPVVNEFRSTKQRFSGTMLTETLPINSCVSPQVSGIKRAHYSGHIPEIQTKKDVACQLDFWGVSPGCPLAGSTLTEDDFLDMSYEEYLEEVHSRGRKQLRISSQETITKPSKFSGN*
	PvCPSF30	Phvul.006G130200.1	MEDSEGVLSFDFEGGLDTAPSAAAAPSGPLVQHDSSAAASAVSNGGPPAPTPSGTEPAAVNVPGRRSFRQTVCRHWLRSLCMKGDACGFLHQYDKARMPVCRFFRLYGECREQDCVYKHTNEDIKECNMYKLGFCPNGPDCRYRHAKSPGPPPPVEEVLQKIQHLYSYNYNSSNKFFQQRGSSYTQQAEKSQLPQGTNSTNQGVTGKPLPAESGNAQPQQQVQQSQQQQVSQNQIQNVANGQPNQASRAATPLPQGISRYFIVKSCNRENLELSVQQGVWATQRSNESKLNEAFDSVENVILIFSVNRTRHFQGCAKMTSRIGGSVAGGNWKYAHGTAHYGRNFSVKWLKLCELSFHKTRHLRNPYNENLPVKISRDCQELEPSIGEQLASLLYLEPDGELMAVSVAAESKREEEKAKGVNPDNGGENPDIVPFEDNEEEEEEESDEEDESFGHGVGPAGQGRGRGRGMMWPPHMPLPRGARPMPGMQGFNPVMMGDGLSYGPVAPDGFGMPDLFSVGPRAFAPYGPRFSGDFGGPPAAMMFRGRPSQPGMFPGGGFGMMMNPGRGPFMGGMGVAGANPPRGGRPVNMPPMFPPPPLPQNTNRLAKRDQRTTDRNDRYGSGSEQGKSQDMLSQSGAPDDDMQYQQGYKANQDDHPAVNNFRNDDSESEDEAPRRSRHGEGKKKRRGPEDVNTNYNH*
	PvFIP37	Phvul.002G107400.1	MASPTHFDDEFDFGGGFGGRHSASKRSSPDYDDEDYDNDPFARKKAQSKAEEASGVTTGMILSLRESLQNCKDTLVTCQNELEAAKSEIQSWHSTLKNEPSVPAGITPDPKMLINHLQTLKTSEESLREQLEKAKKKEAAFIVTFAKREQEITELKSAVWDLKVQLKPPSMQARRLLLDPAVHEEFTRLKNLVEEKDKKVKELQDNIAAMNFTPQSKMGKMLMAKCRTLQEENEEIGNQASEGKIHELALKLALQKSQNSQLRSQFEGLQKHMEGLTNDVERSNETVLMLQDKLEEKDREIQRLKHELQQKNLEDARSDAALTRNDNNETMAGEAAN
	PvHAKAI	Phvul.007G267500.1	MLQIRLSRAPASEGSAGVKQSLLENVTVACPDHLVLADLPVAKGIGAATATSLVKTLGRRSRRQLGERVHFCVRCDFPVAIYGRLSPCEHAFCLDCSRSDSMCYLCDERIQKIQTIKMMEGILICAAPHCLKSFLKKADFESHIQDSHANLLRPNADKEDGNESEAQSVRQSTASDSTARGPQRPVFSPGSNSQQHDLEEKSRRQTPREQPSRQTMQPKPPYYGQHPSDTMSGSVGGGQQGFHQQSFDMQQPPQEPSQFSDRQQAVGPETPFPEYPTMHPAQPSNVPSLVTSNPMLNPPLPFGYPPYPNERAQPFYTAPYDMPRQDSGADIGGDQSSLLGFPQGAPNGPNFPGNYPQPWNSGIGGVPFEQAQGGMVVDPREGKGILAPQPMPLPPPPPPPPNMSHLKQNYYSGELGHDGQGYGWQHDNRDSFGSHG
	PvVIR	Phvul.008G108800.1	MGRPEPCVLFSQTFVHPHLDEYVDEVIFSEPIVITACEFLEQSASSVAQAVSLVGATSPPSFAIEVFVHCEGETRFRRLCQPFLYSQSSSNVLEVEAVVTSHLVVRGSYRSLSLVIYGNTAEDLGQFNIDIDDNALTDLVDSTEGKLEDLPPALHSTNFTIRDSRSSLSVLSIPVPATNIALEVNLFLQLMLKFLEFSDPGDAGHKIVNSVVSAISSYISSDICESISGRYQMWKRSENLEELHGAINEARKELLEVYKVLHRKSRSDSSECSSEANYLEMDVEMLDSKTLVDMFNQYFNFQIHSSCTGDHCLSQREHALLGLSMAYLLCSGRESGFQFVSSGGMEQLAVFFSKDGQNSTTIMLLLLGVIERATRYSVGCEAFLGWWPREDESIPSGISEGYSYLVKLILSKPRHDVASLATYLLHRLRFYEIASRYESAVLSVLENISTVGRVTDVTLNMLSSAEILLRKLLNLINSRGPIEDPSPIARASRSLITGQTDGLLSYKTTSSLISSSSCCFSDCDIDSHLLGLLKERGFLSLSTALLSSSILRTGTGHVMELFMDVTSSVEAVILSFLFSRSGLIFLLQDPELSSTLILALRGGHRGNKENCIPLQYASILISKGFFCSPLEIGMIIEMHLKMANATDSLLSSNPQSEEFLWVVWELSTLSRSDCGRRALLALGNFPEAVSILIEALSSIKESESVGKNSGSSAVNLTIFHSAAEIIEAIVTDSASSSLGSWIGHAMELHRALHFSSPGSNRKDAPSRLLEWIDAGVVYHKHGGIGLMRYAAVLASGGDAQLTST

			SILVSDLTDVENVVGESSSGSDINVMENLGKFISEKSFDGVTLRDSSLAQLTTALRILSFISENPTVAATLYNEGAVIVIYAILVNCRFM<br>LERSSNNYDYLVDEGTECNTTSDLLLERNRELNIVDLLVPSLVLLITLLQKLQEAKEQHRNTKLMNALLRLHREISPKLAACAADLSS<br>RYPDYAIGYGAVCHLIASALAFWPVHGWSPGLFNTLLASVQSSSLLTLGPKETCSLLYLLSDLFPEEDIWLWTSGMPLLTTRRMLGIG<br>TILGPQKERHVNWYLESGHLEKLLGQLVPHLDKIAEIIQNYAISALGVVQDLLRVFVIRISCQNPKYASILIKPVLSSIVHLASESSFPSD<br>TDAYKILRLLDFLVSLLEHPLGKVLLLREGTLQILTKLLDRCFVITDDGKQTPDRSSATCSFNIYSWCLPIFKFIMLLFHSETSHHYPRR<br>HDFKNFEKLSDEDSALILQYILKSCQVLPVGKELLACLTAFKDLASCDEGQMAFGATHLGINSHAYELDPRKGDRNVNYSVSSVAE<br>WRKCPPLLSCWMKLLKSIDDTKEGLSTCAIEAVYALSVGSIQFCMNGDSLNSDRVVALKYLFGISDDMTRSVGFPEENINYILEFSAL<br>LSSKAAMDDCLVTSFSQIPLYQVSESVKSLSLILERPAGSMKLEDAVLPQYDVLGFSNRHQLLENSVEKIDDHLYVGGLGDKFLWEC<br>PEILPDRLTQTNLAAKRKLPSMDGPVRRARGESFQGDISSQNAFSRGPAQSAVSSGTTRRDAFRHRKPNTSRPPSMHVDDYVARERI<br>VEGVTNVISVPRAGSTGGRPPSIHVDEFMARQRERQNPSATVVGEAVGHLKNASPVKPADMEKLNKSKQLKTDLDDDLQGIDIVFD<br>GEESDPDDKLLFPQLDDNIQQPAPVIVEQSSPHSIVEETGSDVVDSGQFSQMGTPLRSNVDENAQSEFSSKISGSRPDMSLTRESSVSS<br>DRKYVEQADDLKNVQVKPSGRYDSAASNTSFPMSLYNNPSSSMQLPADSRMVSQNYLLKNSPQHGGIATGSQGLYDQRFLPNQPP<br>LPPMPPPPTVSPIISHATDSVPSQSTSFVNPQAGTQRPVAFQVQLDYPSPFNNGTTATALASSIPMQDSKYSRTSVSSPGGPNRVAPPLP<br>PTPPPFVSSQYNLSSVKSSGSQPSIYNQTSMGTTELSHSSIASSGARLSSYPNPPMGFSRPASMPLSMFGNAPNQQQTENQPNILQNISV<br>PPASFQSMHSVTQLQPLQPPQLTRPPQPPQLRPPVQALQQLEQGMAVQSNAQVHQINMLQQSQVPSMQTYYQTQQQQFSHEQLQPH<br>VEYTQQPADGQSQQQPDAGLSLHEYFKSPEAIQSLLRDRDKLCQLLEQHPKLMQMLQERLGQL

Supplementary Table S3. Primer sequences of m^{6}A regulatory genes for RT-qPCR

Gene ID	Transcript ID	Forward Primer(5'→3')	Reverse Primer(5'→3')
PvMTA	Phvul.010G102500.1	CTAACTCACCGCTCCAATTCT	CTGCTAACGGACTAACCTTCTC
PvMTB	Phvul.007G073300.1	CAGGACTGATAGCGACGATTAC	CCTCATACCAACTGCTCAACT
PvMTC	Phvul.001G016200.1	ACACCCAAGATGATGCAGTAG	TCAGCGTGAGCAGGAATTAG
PvALKBH1A	Phvul.001G262100.1	CTGGGCTGCAAGGCTATATT	CTTGCATCTCCAGCCATAAGA
PvALKBH1C	Phvul.001G131400.1	AGTTTGTCGGAAACTTGGTCTA	GTCTGAGGATCCCAATCCATAC
PvALKBH6	Phvul.004G131600.1	ACCAGCCTAATCAAGGCATAAT	CAACAGGAGACCCAAGTGATAG
PvALKBH7	Phvul.008G264300.1	TGGCTCCTTGATTCTCATGTC	AGTGATGGAGGTGCGTTTAG
PvALKBH8	Phvul.002G123600.1	GGCCCTGTATAATGGAGTTCAG	AAAGACCGAGGAGGAAGATAGA
PvALKBH9A	Phvul.001G044000.2	ACTCCGAGCCAGAGAATTTG	TCACCCACATCAACAGTAACC
PvALKBH9B	Phvul.006G214800.1	GTAAGGGACGTGTCACAATACA	GGTGGTAATGGATCAACCTCTT
PvALKBH10A	Phvul.001G147800.1	CTTGGACGACGAACAGAAATTG	CACTGTCTGGCGAATCATACT
PvALKBH10B	Phvul.007G168900.1	GGACCTCTCATGCTCTCATTG	GATGCTGACCCTTCTGTTAGG
PvALKBH10C1	Phvul.002G181800.1	CAAGTGATGCTCAACGTCTTTAC	TGCTTGGAGCCTACAGAATG
PvALKBH10C2	Phvul.003G014200.1	CGAATGCCATCATAGACTCCTT	TTACACCTCCTCTGCTGAATTG
PvECT1	Phvul.001G110200.1	CTGGTGGCTGTCCCATATTT	CTGTCCTGCTGCCAATAATCTA
PvECT2	Phvul.002G247000.1	ATGGCTACCGTTGCTAATCC	CTTGGTAGGTTCAGGCATCTC
PvECT3	Phvul.004G080300.1	CCTGGTGACTGTCCTGTATTT	AAGCAACCAGTCCACCTATC
PvECT5	Phvul.010G165400.1	GGCCTGCCCTATATTTCTCTTT	GCCAGAAGTCCACACTCTTATC
PvECT6	Phvul.006G121600.1	CAGAAGGGCCAAGAGGTAATG	TGAACAGCTGGTGAAGCTAAA
PvECT7	Phvul.003G119300.1	ACATCCCTCCAGTCAGTAAGA	GTTGGTGACCTCAGAACTACAC
PvECT8A	Phvul.005G045600.1	GAACTGCCTGGTAAACCAAATG	GAGGAGGGTGAAGACTTTGATAC
PvECT8B	Phvul.004G132700.1	GTGTGGGCAAGTACTCCTAATG	GGGCACTTGCATCCTTTATCT
PvECT11	Phvul.006G218800.1	CTCTTCTCTCCAACCTCTCTTTC	AGTTCCTTTAGGGACGGATTG
PvECT12	Phvul.002G152600.1	TAGCAGAGACTGTCAGGAGTTA	CCCTTGATGAACTGGTCAGTAG
PvCPSF30	Phvul.006G130200.1	AGGTGGTAGGCCGGTTAATA	CCGGAGCCATACCTATCATTTC
PvFIP37	Phvul.002G107400.1	CTTGAAGAGAAGGATCGGGAAA	CTCCAGCCATCGTCTCATTATT
PvHAKAI	Phvul.007G267500.1	TCTCGTAGACAGACACCTAGAG	AACAGAGCCAGACATGGTATC
PvVIR	Phvul.008G108800.1	CTCAAGGACTGTACGACCAAAG	GAATCGGTGGCGTGAGATATTA

Supplementary Table S4. Primer sequences used in this study

Gene ID	Primer Sequence
BK-MTA-F	CATGGAGGCCGAATTCCCGGGGATGGAAACACAATCAGACGG
BK-MTA-R	GCAGGTCGACGGATCCCCGGGTTAACCAATGTCAACATCAA
AD-MTB-F	GGCCAGTGAATTCCACCCGGGTATGGATTCGAGCGACAGTGGT
AD-MTB-R	CCCGTATCGATGCCCACCCGGGTGCTACAACATATTCATTTGTCT
BIFC-N-MTA-F	TCCGTCGACGGTACCCCCGGGATGGAAACACAATCAGACGG
BIFC-N-MTA-R	ACCAGACCCGCCTCCCCCGGGACCAATGTCAACATCAATGG
BIFC-C-MTB-F	GTGGATCCGTCGACGGTACCCCCGGGATGGATTCGAGCGACAGTGGT
BIFC-C-MTB-R	ACCAGACCCGCCTCCCCCGGGACCAATGTCAACATCAATGG
pCV-GFP-EHA+MTA-F	CTGCCCGGGGCCTGG GGTACC ATGGAAACACAATCAGACG
pCV-GFP-EHA+MTA-R	CATGTCGACGCACAG GGTACC ACCAATGTCAACATCAATG
pCV-GFP-MTB-F	CTGCCCGGGGCCTGG GGTACC ATGGATTCGAGCGACAGTG
pCV-GFP-MTB-R	CATGTCGACGCACAG GGTACC CAACATATTCATTTGTCTG
OE-*PvMTA*-F	AACACGGGGGACTTTGCAACATGGAAACACAATCAGACGGTAATGAGGAC
OE-*PvMTA*-R	TAATCAAACCACCAATGTCAACATCAATGGCCATTGACTTATC
IP-qPCR-4568-F	AGCGCTATCTAAGGGACTACA
IP-qPCR-4568-R	TGTTGAGAACTCCCAAATCCC
qBCMV-DY9-CP-F	TCAACACAAGAGCAACAAAGATG
qBCMV-DY9-CP-R	GTCAATGCACCACACCATAAAG
NtubI-qPCR-F	TCCAGGACAAGGAGGGTATCC
NtubI-qPCR-R	GTCAGCCAAGGTCCTTCCATCC
Act11-qPCR-F	TGCATACGTTGGTGATGAGG
Act11-qPCR-R	AGCCTTGGGGTTAAGAGGAG
V2-MTA-F	CCAGATCAGGATCTCTCGAG TGCAGGATGGATGTCTCTTGGT
V2-MTA-R	AATCCTCTGCATGAGGATCC ACCAATGTCAACATCAATGGCC
V2-MTB-F	CCAGATCAGGATCTCTCGAG ACAGGTAGCGGGTCAAACAGAA
V2-MTB-R	AATCCTCTGCATGAGGATCC CAACATATTCATTTGTCTGTAA
V3-MTA-F	ACACCAAAGGGAAGC CTCGAG ATCGAAACACAATCAGACGGTAATG
V3-MTA-R	TTGCTTAGCTGGGGC CCCGGG ACCAATGTCAACATCAATGGCCATT
V3-MTB-F	ACACCAAAGGGAAGC CTCGAG ATGGATTCGAGCGACAGTGGTCGCG
V3-MTB-R	TTGCTTAGCTGGGGC CCCGGG CAACATATTCATTTGTCTGTAAGAT
