# Supplementary figures and images for: Characterization of the m6A Regulatory Gene Family in Phaseolus vulgaris L. and Functional Analysis of PvMTA in Response to BCMV Infection
Source: Int J Mol Sci. 2025 Mar 19;26(6):2748. doi: 10.3390/ijms26062748 (PMC11942742; doi:10.3390/ijms26062748)

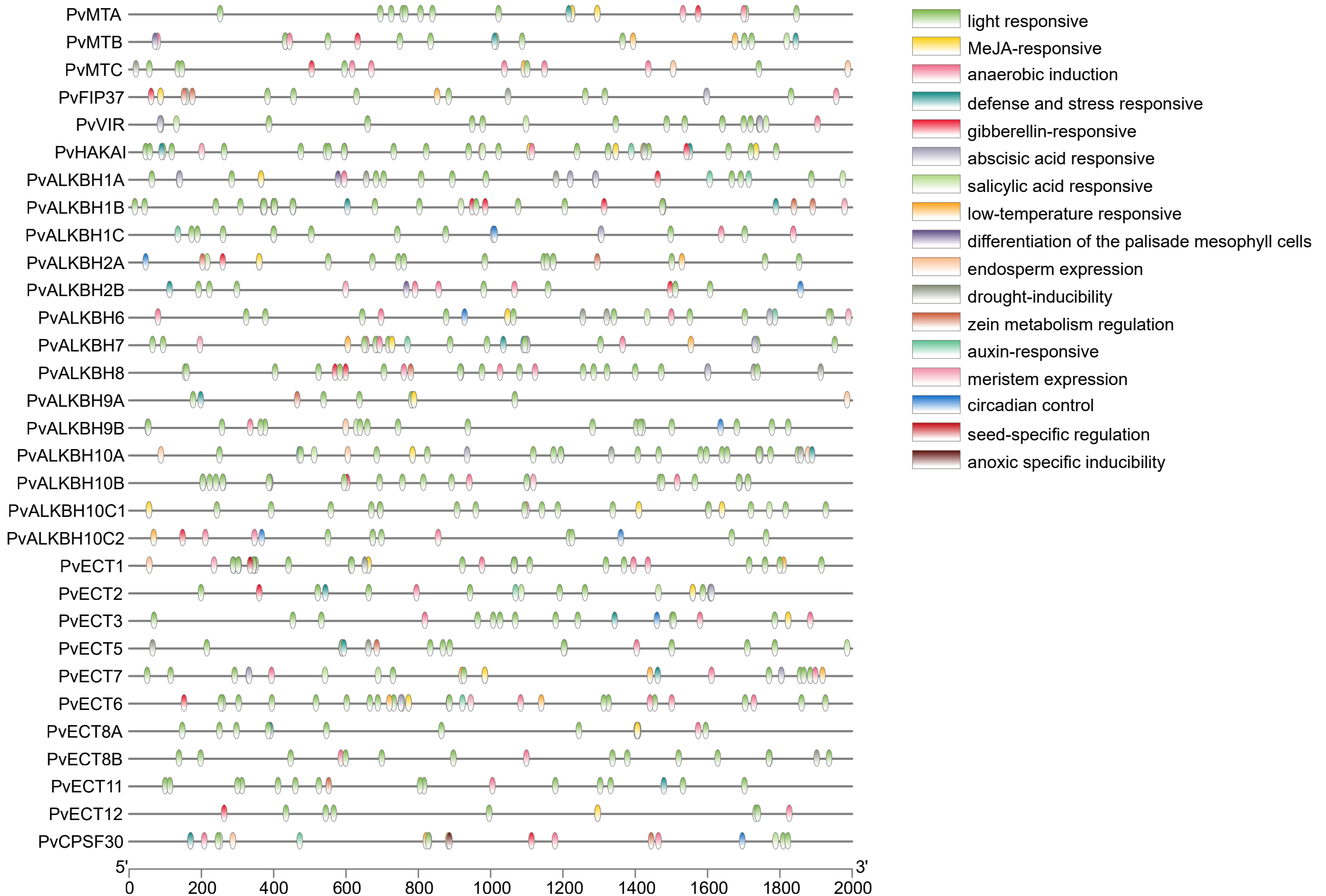

Supplement: Supplementary file 1 [file ijms-26-02748-s001.zip › Figure S1.pdf]

Figure S2

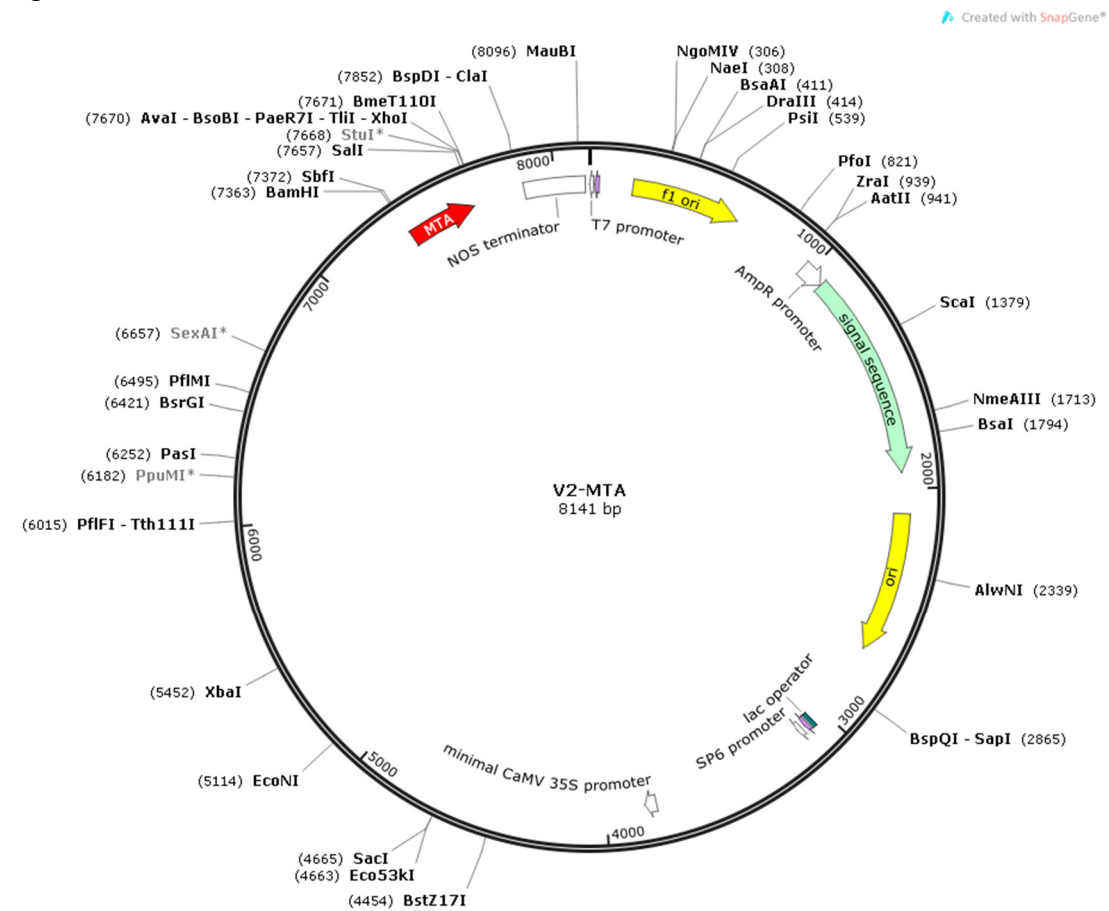

The map of V2-MTA (the desired gene expression recombinant vector)

Supplement: Supplementary file 1 [file ijms-26-02748-s001.zip › Figure S2.pdf]
